# PIK‐III‐Mediated Elevation of Thiamine Re‐Sensitises Renal Cell Carcinoma to Cuproptosis via Activating PDHA1

**DOI:** 10.1111/cpr.70101

**Published:** 2025-07-31

**Authors:** Dongdong Xie, Yu Wang, Wenjie Cheng, Minbo Yan, Kunyu Li, Xiang Wu, Jiaqing Wu, Zhuangzhuang Zhang, Yingbo Dai

**Affiliations:** ^1^ Department of Urology The Fifth Affiliated Hospital of Sun Yat‐sen University Zhuhai China; ^2^ Guangdong Provincial Engineering Research Center of Molecular Imaging The Fifth Affiliated Hospital, Sun Yat‐sen University Zhuhai China; ^3^ Guangdong‐Hong Kong‐Macao University Joint Laboratory of Interventional Medicine The Fifth Affiliated Hospital, Sun Yat‐sen University Zhuhai China; ^4^ Department of Kidney Transplantation The Third Affiliated Hospital of Sun Yat‐sen University Guangzhou China

**Keywords:** ccRCC, cuproptosis, PIK‐III, thiamine metabolism, Warburg effect

## Abstract

Cuproptosis, a copper‐dependent cell death mechanism driven by tricarboxylic acid (TCA) cycle collapse, shows limited efficacy in hypoxic or glycolytic renal cell carcinoma (RCC). Here, through systematic screening of 688 glycolysis inhibitors combined with elesclomol (ES), we identified PIK‐III as a potent cuproptosis sensitiser. Multi‐omics analysis revealed that PIK‐III restores sensitivity by rewiring thiamine metabolism. Mechanistically, PIK‐III induces macropinocytosis, enabling thiamine uptake to replenish thiamine pyrophosphate (TPP), which activates pyruvate dehydrogenase E1‐alpha 1 (PDHA1) and redirects pyruvate into the TCA cycle. Concurrently, ES‐induced DLAT oligomerisation disrupts TCA flux, creating a metabolic crisis. In vivo, PIK‐III synergises with ES to suppress tumour growth in xenograft and patient‐derived models without systemic toxicity. Our work uncovers a metabolic vulnerability in cuproptosis‐resistant RCC and positions PIK‐III as a therapeutic candidate to overcome resistance via dual targeting of thiamine transport and mitochondrial dysfunction.

## Graphical abstract

PIK‐III enhancing the efficacy of cuproptosis to kill renal cancer cells through dysregulating thiamine metabolism and dephosphorylation of pyruvate dehydrogenase complex E1 (PDHA1), providing a potential option for treatment of cuproptosis‐resistant renal cancer by the combination of PIK‐III and elesclomol.

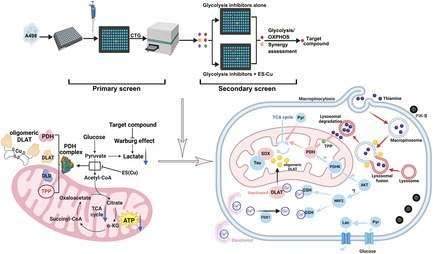

## 1 Introduction

The most recent global epidemiological data on renal cell carcinoma (RCC) indicate that there were 431,288 new cases diagnosed worldwide in 2020 [1]. According to the American Cancer Society, the incidence rate of renal cancer in the United States is increasing, standing at 17.3 per 100,000 population, yet there has been no significant reduction in mortality rates despite advancements in medical technology [2, 3, 4]. Clear cell RCC (ccRCC), which constitutes approximately 90% of all renal cancer cases [1, 2, 3, 4, 5, 6], underscores the urgent clinical need for effective adjuvant therapies to diminish ccRCC‐related fatalities.

Copper, an essential trace element, is integral to cellular homeostasis, acting as a cofactor for enzymes involved in mitochondrial respiration and biosynthesis [7, 8, 9, 10]. The reliance of tumour cells on copper for proliferation and metastasis exceeds that of normal tissue, highlighting the potential of copper level regulation in cancer treatment [11, 12]. Disruptions in copper metabolism, however, can be cytotoxic, with an excess leading to redox imbalances [7, 8]. Cuproptosis, a mechanism of copper disruption‐induced cytotoxicity [7, 8, 13], primarily involves the oligomerisation of dihydrolipoamide S‐acetyltransferase (DLAT), a key component of the pyruvate dehydrogenase (PDH) complex. The PDH complex's activity, essential for acetyl‐coenzyme A synthesis, is contingent upon the lipoylation of DLAT, which transfers the hydroxyethyl group from thiamine pyrophosphate (TPP) to coenzyme A [14]. The execution of cuproptosis also depends on ferric redox protein 1 (FDX1), which can reduce Cu^2+^ to the more cytotoxic Cu^+^ upon elesclomol (ES) treatment [7, 8, 13, 15]. FDX1 is also crucial for the lipoylation of mitochondrial proteins [15]. Elesclomol in conjunction with Cu^2+^ (ES‐Cu) induces DLAT oligomerisation within mitochondria by modulating FDX1 levels, triggering cuproptosis [15].

The traditional energy source for cancer cells, ATP generated through aerobic glycolysis, is exemplified by the Warburg effect, characterised by elevated lactic acid production [16]. Tsvetkov et al. demonstrated that ES‐Cu treatment significantly increased tricarboxylic acid (TCA) cycle metabolites in ABC1 cells, whereas no substantial changes were observed in the cuproptosis‐resistant A549 cells [17]. The sensitivity of tumour cells dependent on anaerobic/aerobic glycolysis to ES was markedly lower than that of cells relying on aerobic respiration, suggesting that tumour cells favouring the Warburg effect may be resistant to ES‐induced cuproptosis [15, 17].

Von Hippel–Lindau (VHL), a tumour suppressor acting as an E3 ubiquitin ligase, mediates the proteasomal degradation of hypoxia‐inducible factor (HIF), a pivotal regulator of hypoxia and aerobic glycolysis in tumours [18, 19, 20, 21, 22]. Prior research indicates that cuproptosis‐resistant patients exhibit poorer survival and clinicopathological outcomes, including advanced tumour stage, grading, and metastasis, compared to sensitive patients [23]. These findings underscore the intricate relationship between cuproptosis, hypoxia, and glycolysis, and suggest the existence of cuproptosis resistance in ccRCC patients with adverse prognoses [15, 18, 19, 20, 21, 22, 23]. Xing and colleagues reported that pyruvate kinase M (PKM) shifts cellular glucose metabolism from aerobic glycolysis to the TCA cycle and oxidative phosphorylation (OXPHOS) in ARID1A‐deficient hepatoma carcinoma cells, indicating that a preference for aerobic glycolysis may contribute to cuproptosis resistance [24].

Our investigation into the interplay among ES, the Warburg effect, and cuproptosis aims to explore the potential expansion of ES's clinical applications [25, 26]. Given the potential resistance of ccRCC to ES‐induced cuproptosis, this study seeks to identify compounds that can enhance the sensitivity of ccRCC to cuproptosis, thereby paving the way for clinical application and deepening our understanding of the mechanisms underlying cuproptosis sensitisation.

## 2 Materials and Methods

### 2.1 Cell Lines and Culture

Renal cancer cell lines A498, ACHN, OSRC‐2, and 786‐O were procured from Procell. Additionally, the human renal cancer cell line UMRC6, human renal cortical proximal tubular epithelial cells (HK2), human normal hepatocytes (L02), and human umbilical vein endothelial cells (HUVEC) were sourced from the Molecular Imaging Center of the Fifth Affiliated Hospital of Sun Yat‐sen University, accompanied by professional STR certification obtained in July 2022. Cell culture media, MEM (PM150410, Procell) for A498 and ACHN, 1640 (C11875500BT, GIBCO) for OSRC‐2, 786‐O, and UMRC6, and DMEM (C11995500BT, GIBCO) for L02 and HUVEC, were supplemented with 10% fetal bovine serum (FBSF, Moregate) and antibiotics (100 U/mL Penicillin and 100 μg/mL Streptomycin, 15140122, GIBCO). HK2 cells were maintained in DMEM/F12 medium (C11330500BT, GIBCO). Culture conditions for all cell lines were a humidified incubator with 5% CO_2_ at 37°C. An anoxic environment of 1% O_2_, 5% CO_2_, and 94% N_2_ was maintained during the drug screening phase to simulate hypoxic conditions.

### 2.2 Cell Viability Assay

Cell viability assays were conducted using cells seeded in 96‐well plates at a density of 4000–5000 cells per well to assess cell number following drug treatments. Each treatment was performed in triplicate, and compounds were introduced 48 h post‐seeding. In chemo‐recovery assays, regulators of cell death were added 8 h prior to the induction of cell death by specific compounds. Cell viability was assessed 24 or 48 h post‐drug addition using the CellTiter‐Glo assay (G7571, Promega), as per the manufacturer's instructions.

### 2.3 Xenograft Tumour Model

BALB/c nude mice, aged 4 weeks and obtained from Beijing Spectrum Biotechnology Co. Ltd., were used for in vivo experiments. Mice were subcutaneously injected with 5 million OSRC‐2 cells in the left upper limb and monitored for tumour formation for 14 days prior to drug administration. Tumour volume was calculated using the formula: (maximum diameter × (minimum diameter)^2^)/2. All mice were housed in a pathogen‐free environment at the Molecular Imaging Centre of the Fifth Affiliated Hospital of Sun Yat‐sen University. The Animal Care and Use Committee of the centre approved all experimental procedures involving mice.

### 2.4 Compounds

ES (STA‐4783) was sourced from Selleck, and a library of 688 aerobic glycolysis inhibitors for screening was obtained from Selleck Chemicals (L2000\L8700) and MCE Chem (HY‐L058). This library includes FDA‐approved small molecule inhibitors, chemotherapeutic agents, and other compounds. All compounds were prepared in DMSO at a concentration of 10 mM. PIK‐III and POMHEX were also dissolved in DMSO at a concentration of 10 mM. Screened compounds were validated in A498, ACHN, and OSRC‐2 renal carcinoma cell lines following a 24‐h exposure. A comprehensive list of other compounds used in the study is provided in the Supporting Information.

### 2.5 Drug Screening Procedure

For the initial compound screen, A498 cells were plated in black 96‐well plates at a density of 5000 cells/well. After a 48‐h incubation, cells were treated with a combination of 10 μM aerobic glycolysis inhibitors and 40 nM ES with 1 μM Cu^2+^. Cell viability was measured 24 h post‐treatment using the CellTiter‐Glo Luminescent Cell Viability Assay, and fluorescence was read using a BioTek Synergy HT microplate reader. Compounds demonstrating a synergistic effect (*Z*‐score normalised and identified by a *Z*‐score greater than 1) were advanced to the second round of screening. In the second round, the inhibitory effect of single and dual treatments was compared, and compounds with greater than 50% inhibition and a *p*‐value less than 0.05 were selected for further validation in A498, ACHN, and OSRC‐2 cell lines. The Synergy Finder drug synergy assessment model was utilised to analyse the synergistic effects of the target compounds, identifying those with the highest synergy indices for further study.

## 3 Results

### 3.1 Hypoxia and Aerobic Glycolysis Drive Cuproptosis Resistance in ccRCC

**FIGURE 1:**
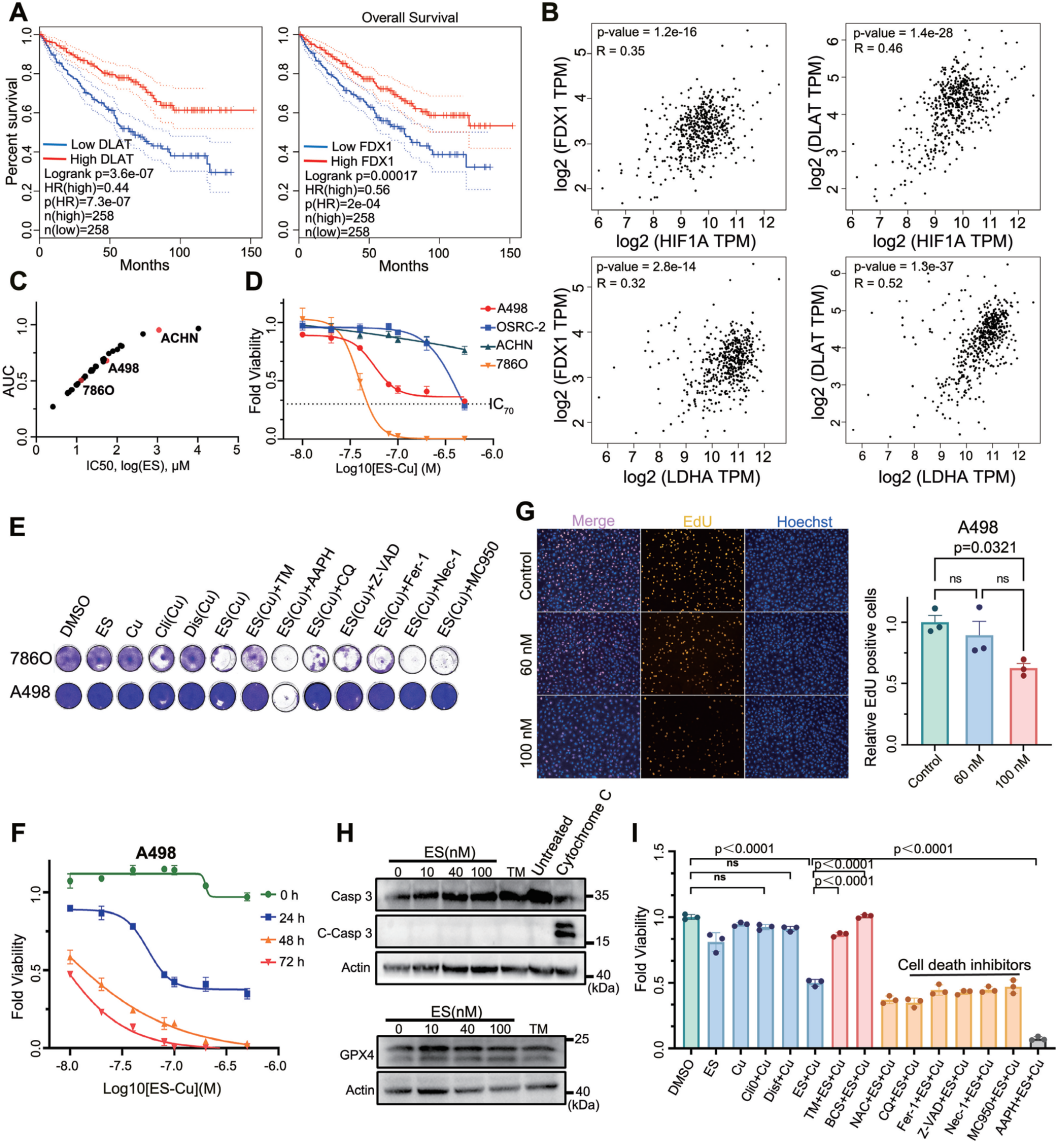
Validation of cuproptosis and screening of resistant cell lines in renal cancer. (A) Kaplan–Meier curves depict overall survival based on DLAT and FDX1 expression levels, analysed using GEPIA with TCGA and GTEx data. (B) mRNA level correlation between cuproptosis‐related genes (DLAT or FDX1) and aerobic glycolysis genes (HIF1α or LDHA) in renal cancer tissues, with TPM values indicating expression levels. (C) Utilisation of the GDSC database to screen for relative resistance to elesclomol among cell lines, with AUC representing the area under the drug‐time curve. (D) Sensitivity differences to elesclomol across renal cancer cell lines. (E) Fraction survival assay comparing the cuproptosis induction potency of various copper ion carriers. (F) Time and concentration gradients of cuproptosis induction in A498 cells by ES‐Cu. (G) EdU staining to assess cell proliferation after treatment with different elesclomol concentrations. (H) Immunoblotting for caspase3 and GPX4 in A498 cells post‐ES‐Cu treatment. (I) Rescue experiments using copper ion chelators and inhibitors of other cell death pathways on cuproptosis in A498 cells. (*n* = 3, data are mean ± SD, One way ANOVA).

Since the discovery of cuproptosis, its potential clinical applications have been extensively explored. Analysis of the TCGA database indicates that elevated expression levels of FDX1 and DLAT are associated with improved patient prognoses (Figure 1A). Hypoxia and increased aerobic glycolysis are implicated in cuproptosis resistance, with significant correlations observed between the expression of hypoxia/Warburg effect‐related genes (HIF‐1α, LDHA) and FDX1/DLAT in RCC tumour cells (Figure 1B). This suggests a link between the hypoxia/Warburg effect and cuproptosis resistance in RCC. To further understand this resistance and identify novel therapeutic agents, we utilised the Genomics of Drug Sensitivity in Cancer (GDSC) database to screen renal cancer cell lines for cuproptosis sensitivity (Figure 1C). In vitro validation confirmed that ACHN, OSRC‐2, and A498 cells exhibit resistance to cuproptosis, while 786O cells display sensitivity (Figures 1D,E and S1A). The purpose of using IC_70_ is to distinguish the two ccRCC cell lines; we can well distinguish A498 and 786O by using IC_70_ as a criterion and define them as cuproptosis resistant and sensitive cell lines. The resistant non‐ccRCC cell lines will be selected for validation experiments in our subsequent experiments. When the ES concentration reached 100 nM, ES‐Cu had a certain inhibitory effect on the proliferation of A498 renal cancer cells; thus, the subsequent cell viability assay controlled the concentration of ES below 60 nM to exclude the effect of cell proliferation (Figure 1F,G). ES‐induced cytotoxicity was significantly enhanced by the addition of copper ions, highlighting the importance of copper in cuproptosis induction (Figure S1B). To confirm whether apoptosis or ferroptosis brought about by a short period of ES‐Cu treatment occurred, we examined the levels of cleaved caspase3 and GPX4, which showed no significant changes in both (Figure 1H). Notably, other copper ion carriers such as clioquinol and disulfiram did not replicate this effect, emphasising the specificity of ES in inducing cuproptosis. The use of N‐Acetylcysteine (NAC) and other inhibitors did not mitigate cuproptosis, suggesting a unique mechanism of action (Figure 1E,I). It further illustrated that ES‐induced cuproptosis distinguishes itself from other types of cell death modes by intracellular copper ion toxicity.

### 3.2 Systematic Screening Identifies PIK‐III Enhancing the Efficacy of ES‐Cu Induced Cuproptosis in ccRCC

**FIGURE 2:**
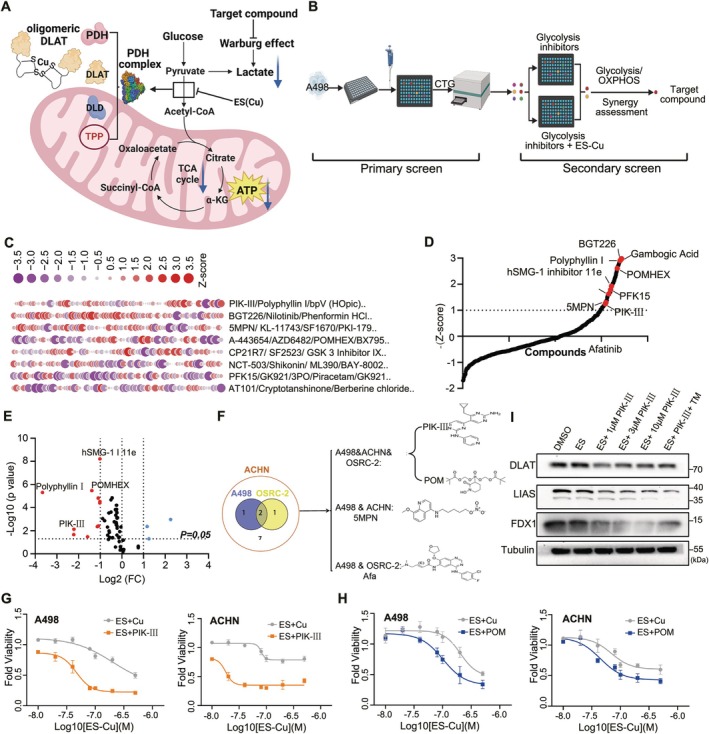
Target compound screening for enhanced cuproptosis sensitization. (A) Schematic representation of the mechanism by which inhibiting the Warburg effect sensitises cells to cuproptosis. DLD, dihydrolipoyl dehydrogenase. (B) Initial screening pattern using the molecular library in combination with ES‐Cu against A498 cell viability (*Z*‐score < −1). Inhibitory effect of the preliminary screened compounds combined with ES‐Cu versus the glycolysis inhibitors alone (10 μM) was tested as the secondary screening, and the final target compounds were selected after the analysis of the compound synergism and ratio of glycolysis/oxidative phosphorylation. (C, D) Schematic of the initial screening process, yielding 58 compounds that met the criteria. (E) Schematic of the initial screening process, yielding 58 compounds that met the criteria (*p* < 0.05, FC‐value < 0.5, two tailed unpaired *t*‐test). (F) Validation of 11 compounds from the secondary screen in A498, OSRC‐2, and ACHN cell lines. POMHEX (POM), Afatinib (Afa). (G, H) Sensitization effects of PIK‐III and POMHEX combinations under hypoxic conditions in A498 and ACHN cells. (*n* = 3, data are mean ± SD). (I) Changes in cuproptosis markers in A498 cells following PIK‐III treatment at varying concentrations. (J) Oligomycin (OM), FCCP, and rotenone (ROT) and antimycin a (AA) were added sequentially into A498 cells treated with ES(Cu) and PIK‐III for 12 h. OCR was assessed by seahorse flux analyser. (K) while glucose (GLU), oligomycin and 2‐deoxy‐D‐glucose (2‐DG) were added sequentially into A498 cells treated with ES(Cu) and PIK‐III for 12 h. ECAR was assessed by seahorse flux analyser.

We conducted a comprehensive screen of 688 FDA‐approved aerobic glycolysis inhibitors to identify novel agents that could potentiate cuproptosis in renal cancer cells. A498 is a typical ccRCC cell line accompanied by VHL mutations and abnormal activation of the HIF pathway. It is thus a preferred strong warburg effector cell model for screening efforts of aerobic glycolysis inhibitors. There are fewer reports in the ACHN cell line related to warburg effects, and the metabolic phenotype is dependent on upstream signalling: including the PI3K/AKT/mTOR pathway and MYC overexpression. The uncontrollable factors thus led us to discard the more resistant non‐ccRCC cell line, but we also added non‐ccRCC for validation in subsequent experiments. Utilising A498 cells, we evaluated the effects of these inhibitors in combination with ES and copper ions (Cu), comparing their impact on cell viability to that of ES‐Cu treatment alone (Figure 2A,B). Compounds that demonstrated a normalised cell viability *Z*‐score of less than −1 were selected for further analysis, yielding an initial set of 58 candidate compounds (Figure 2C,D) [26, 27, 28]. In a secondary screen, we applied stringent criteria, including a fold change (FC) of less than 0.5 and a *p*‐value below 0.05, identifying 11 compounds with significant inhibitory effects (Figure 2E). Among these, PIK‐III and POMHEX, when combined with ES‐Cu, significantly reduced cell viability by over 50% in three cuproptosis‐resistant ccRCC cell lines: A498, ACHN, and OSRC‐2 (Figure 2F). ACHN represents a metastatic model of renal cancer, a mixed metabolic pattern, high aggressiveness, and a mandatory cell line for non‐renal clear cell carcinoma in renal cancer therapeutic drug screening. Given hypoxia is a known inducer of cuproptosis resistance [15, 29], we exposed A498 and ACHN cells to a hypoxic environment followed by treatment with the top 7 inhibitors from the secondary screen. Using the IC_10_ values determined for each inhibitor (Figure S1C–I, Table S1), we assessed their effects in combination with varying concentrations of ES on A498 and ACHN cells (Figures 2G,H and S2A–E) [30]. PIK‐III emerged as the most effective at re‐sensitising ES‐Cu‐resistant cells to cuproptosis under hypoxic conditions (Figure 2G,H). Additionally, PIK‐III treatment led to a notable decrease in cuproptosis‐related markers LIAS and FDX1, with the latter's downregulation being partially reversible by tetrathiomolybdate (TM) (Figure 2I). The synergistic interaction between ES‐Cu and PIK‐III was further analysed using the Synergy Finder platform, confirming a robust synergistic effect compared to POMHEX (Figure S3A,B,E). These results suggest that PIK‐III re‐sensitises tumour cell to ES‐induced cuproptosis only by inhibition of glycolysis but not oxidative phosphorylation in the condition of hypoxia. Evaluation of the toxicity of ES in combination with glycolysis inhibitors on normal cells in vitro showed a favourable biosafety profile (Figure S3C,D). Finally, we assessed the effects of PIK‐III on mitochondrial functions including oxygen consumption rate (OCR) and extracellular acidification rate (ECAR) using Seahorse Flux Analyser. Two micrometres PIK‐III alone did induced slight rise in mitochondrial respiration/glycolysis (Figure 2J,K). Additionally, ES(Cu) also induced a significant decrease in OCR when used alone. And from the results of OCR, ES alone or in combination with PIK‐III significantly decreased the mitochondrial spare respiratory capacity (Figure 2J). In the measurement of ECAR, the increase of ECAR was significantly limited in the ES and ES+PIK‐III groups after the addition of oligomycin (OM), which indicated that the compensatory capacity of glycolysis was insufficient, and the cellular adaptation to energy metabolism was decreased (Figure 2K). The synergistic effect of ES and PIK‐III had the strongest inhibitory effect on mitochondria and glycolysis, which may lead to cellular energetic crisis. However, the inhibitory effect on ECAR was significantly higher than OCR under the combination, indicating that PIK‐III significantly inhibited anaerobic respiration.

### 3.3 Multi‐Omics Links Thiamine Metabolism to Cuproptosis Sensitivity

To delineate the molecular mechanisms underlying PIK‐III‐induced re‐sensitisation to cuproptosis, we conducted a multi‐omics analysis on RCC cells. A498 cells were treated with either normal culture medium (negative control, NC), 40 nM ES, 2 μM PIK‐III, or a combination of 40 nM ES and 2 μM PIK‐III, each treatment being accompanied by 1 μM Cu^2+^ for 8 h. Post‐treatment, mRNA and metabolites were extracted from the cells and subjected to both transcriptome and untargeted metabolome sequencing analyses.

**FIGURE 3:**
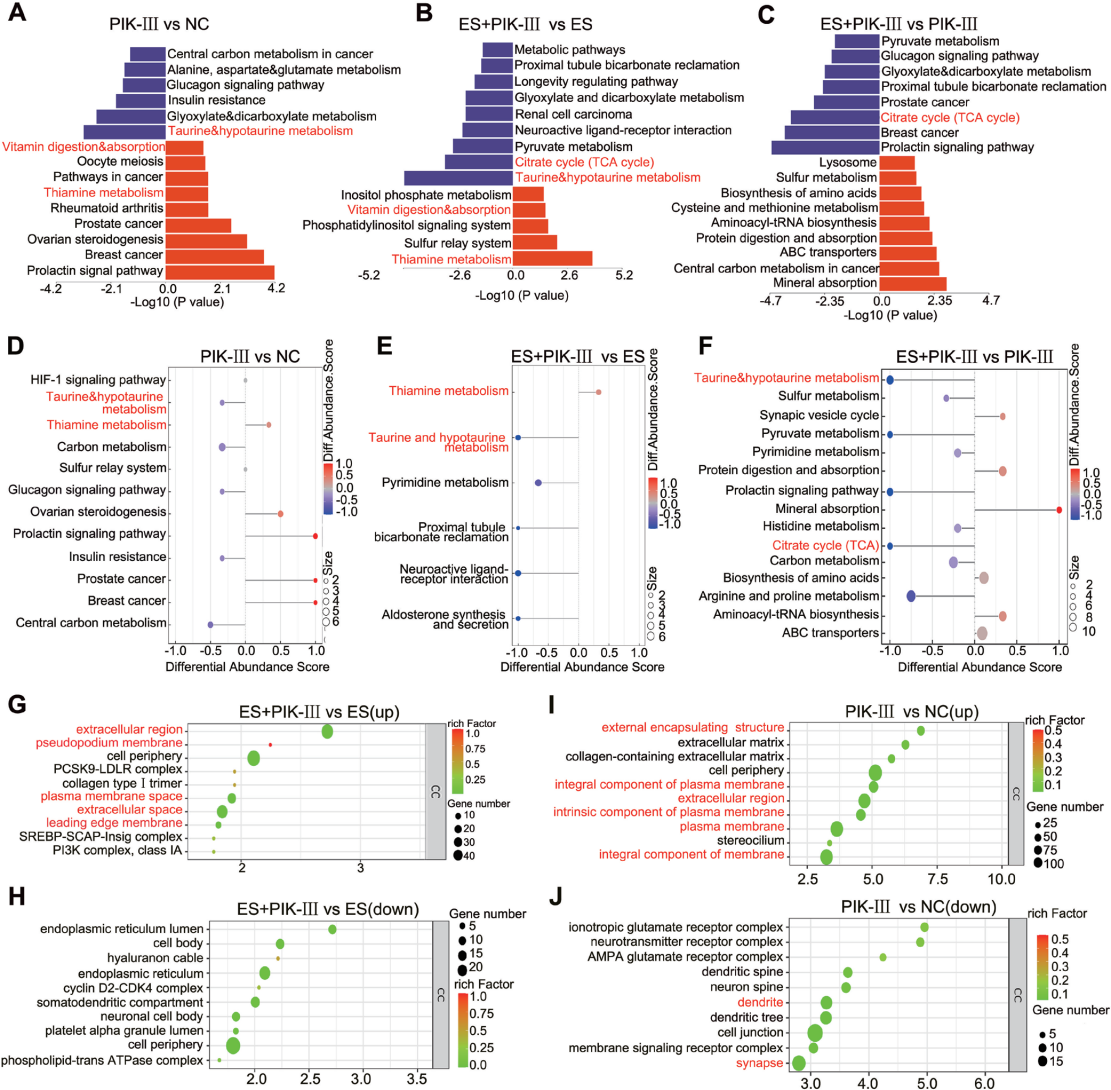
Role of thiamine metabolism in PIK‐III mediated sensitization to cuproptosis. (A–C) Metabolite levels in A498 cells treated with ES, PIK‐III, or ES+PIK‐III, detected by liquid chromatography mass spectrometry. The results of differential metabolite (*n* = 6, OPLS‐DA VIP > 1, *p* value < 0.05) detection were plotted as a butterfly plot, and the blue vertical coordinate is the pathway that the down‐regulated differential metabolites are enriched into, or the red is up‐regulated pathway. (D, F) Differential abundance scores of metabolic pathways enriched between treatment groups. (G, H) Transcriptome differential genes of ES+PIK‐III versus ES group. (I, J) Transcriptome differential genes of PIK‐III versus NC group. The false discovery rate adjusted *p*‐value cutoff was 0.05.

Our analysis anticipated that the combined ES+PIK‐III treatment would redirect ATP production from glycolysis towards oxidative phosphorylation via the TCA cycle. However, a comparative metabolite analysis unexpectedly revealed a significant decrease in taurine and TCA cycle‐related metabolites in the ES+PIK‐III group relative to the ES‐only group, indicative of intracellular oxidative stress and TCA cycle disruption (Figure 3A–C and Figures S4A–D). Metabolic pathway analysis via the Kyoto Encyclopedia of Genes and Genomes (KEGG) highlighted the enrichment of taurine and thiamine metabolism pathways following PIK‐III treatment (Figure 3D–F). Furthermore, we observed a potential for excess thiamine to accumulate intracellularly as thiamine monophosphate (TMP) and TPP, influencing cuproptosis sensitivity (Figures S4A–D and S5A,B).

Transcriptome data analysis also indicated an enrichment of the PI3K‐AKT signalling pathway in response to ES+PIK‐III treatment compared to single treatments (Figure S5C,D). Gene Ontology (GO) enrichment analysis of the transcriptomic data further revealed significant enrichment in cellular components associated with the ‘extracellular region,’ ‘pseudopodium membrane,’ and ‘extracellular space’ in the ES+PIK‐III group, suggesting alterations in the plasma membrane that may facilitate the uptake of extracellular substances (Figure 3G,H). These findings, in conjunction with the observed changes in membrane‐related GO cellular components, imply a role for the plasma membrane in thiamine metabolism and the re‐sensitization to cuproptosis induced by PIK‐III (Figure 3I,J). These results suggest that upregulation of thiamine metabolism may account for the re‐sensitization to cuproptosis induced by PIK‐III. In addition, the occurrence of dramatic change of plasma membrane implies its roles in thiamine metabolism.

### 3.4 PIK‐III Enhances Thiamine Uptake via Macropinocytosis in RCC Cells

**FIGURE 4:**
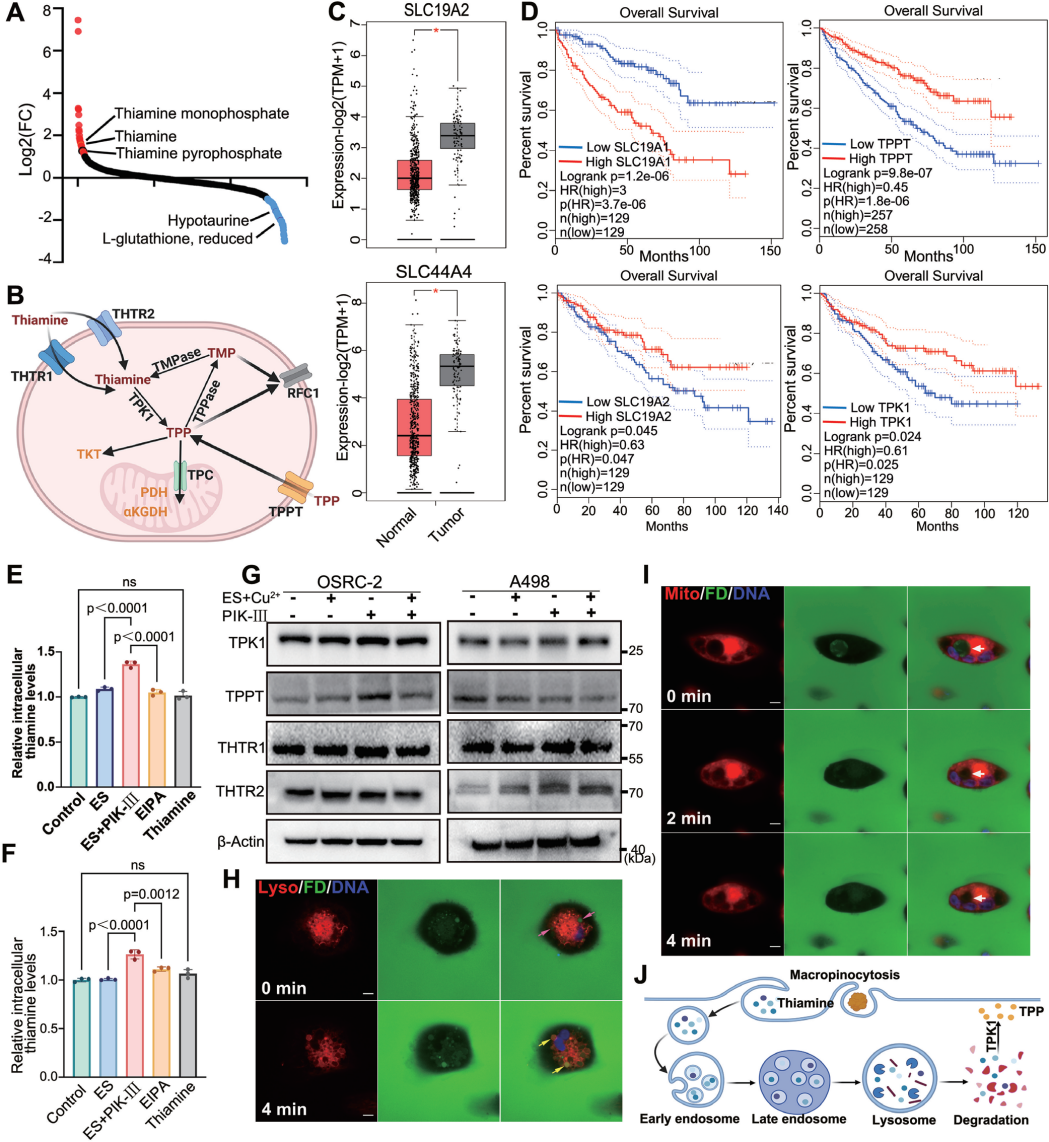
PIK‐III enhances thiamine uptake by inducing macropinocytosis in renal cancer cells. (A) Metabolomics analysis of differential metabolites in ES+PIK‐III versus ES group. (B) Summary of thiamine metabolism pathways and related enzymes. TMPase (thiamine monophosphatase), TPPase (thiamine pyrophosphatase), RFC1 (reduced folate carrier 1), THTR1/2 (Thiamine transporter), TPC (thiamine pyrophosphate carrier), TPPT (Thiamine Pyrophosphate Transporter) and TKT (transketolase). (C) Expression of SLC19A2 and SLC44A4 in KIRC and normal tissues, analysed by GEPIA2. Number of tumour tissues is 523 (Red box), number of normal tissue is 100 (Black box). (D) Kaplan–Meier survival curves based on expression levels of thiamine metabolism‐related genes of SLC19A1, SLC19A2, TPPT(SLC44A4) and TPK1. (E, F) Intracellular thiamine levels in OSRC‐2 and A498 cells under various treatments. *n* = 3, data are means± SD, one‐way ANOVA. (G) Changes in thiamine metabolism‐related channels and enzymes post‐treatment. (H) Macropinosome uptake and lysosomal localization in OSRC‐2 cells following ES and PIK‐III treatment. Unfused macropinosomes are labelled in pink and fused macropinosomes are labelled yellow. (I) Time‐lapse imaging of macropinocytosis and mitochondrial localization in OSRC‐2 cells after treatment of ES and PIK‐III. Macropinosomes are marked by white arrows. Scale bar = 5 μm. (J) Schematic summarising the relationship between macropinocytosis and thiamine uptake in cancer cells.

Metabolic profiling has revealed that treatment with PIK‐III leads to elevated intracellular levels of thiamine and its phosphorylated derivatives in RCC cells (Figures 4A and S5A,B). The mechanism behind this increase remains to be elucidated. In eukaryotic cells, thiamine uptake is predominantly facilitated by the transporters SLC19A2 (THTR1) and SLC19A3 (THTR2), with THTR1 being the principal mediator of thiamine uptake [31, 32]. TPK1, the rate‐limiting enzyme in TPP production, phosphorylates thiamine, while SLC44A4 (TPPT) is implicated in the uptake of extracellular TPP. Conversely, SLC19A1 (RFC1) functions as the primary efflux carrier for thiamine and its metabolites (Figure 4B) [31, 32].

To explore the basis of thiamine imbalance in RCC cells, we examined THTR1 and TPPT expression levels in RCC samples using the TCGA database. Our findings indicated that the expression of THTR1 and TPPT was significantly downregulated in RCC tissues compared to adjacent normal renal tissues, suggesting a reduced capacity for thiamine uptake (Figure 4C). Intriguingly, diminished expression of THTR1, TPPT, and TPK1 was correlated with poorer prognoses in RCC patients, while higher RFC1 expression levels were associated with adverse outcomes (Figure 4D). These observations point towards a ‘thiamine‐deprived’ phenotype in renal cancer, where enhanced thiamine uptake may promote a shift towards glycolysis over oxidative phosphorylation [31].

Bioinformatics analysis, including GO enrichment, postulated that PIK‐III might increase extracellular thiamine uptake by modulating plasma membrane properties. Subsequent microscopic analysis of PIK‐III‐treated RCC cells disclosed the presence of distinct vacuole‐like structures (Figure S6A). Co‐staining with LC3 and Nile Red excluded the presence of autophagic markers or lipids within these vacuoles (Figure S6B), prompting the hypothesis that these structures might be macropinosomes responsible for enhanced thiamine uptake in RCC [33]. This hypothesis is supported by GO enrichment analysis, which indicated upregulation of terms associated with macropinocytosis, such as ‘extracellular region’ and ‘pseudopodium membrane’ (Figure 3G,H).

Measurement of intracellular thiamine levels in PIK‐III‐treated cancer cells confirmed an increase, which was not significantly affected by the addition of exogenous thiamine to the culture medium. Moreover, this increase was significantly mitigated by the macropinocytosis inhibitor EIPA (Figure 4E,F). Notably, the expression of thiamine‐related transport channels did not appreciably change in response to PIK‐III treatment, indicating that the observed increase in intracellular thiamine does not involve alterations in these channels (Figure 4G).

Further investigation using FITC‐dextran and lysotracker co‐staining on PIK‐III‐treated cancer cells provided visual evidence of vacuole formation and subsequent lysosomal fusion (Figure 4H). The release of vacuolar content into the cytoplasm over time was validated using co‐staining of FITC‐dextran with mitotracker (Figures 4I and S7A). Collectively, these findings suggest that PIK‐III‐mediated macropinocytosis is likely responsible for the observed increase in intracellular thiamine levels (Figure 4J).

### 3.5 TPP‐Dependent PDHA1 Activation Restores Mitochondrial Pyruvate Flux

**FIGURE 5:**
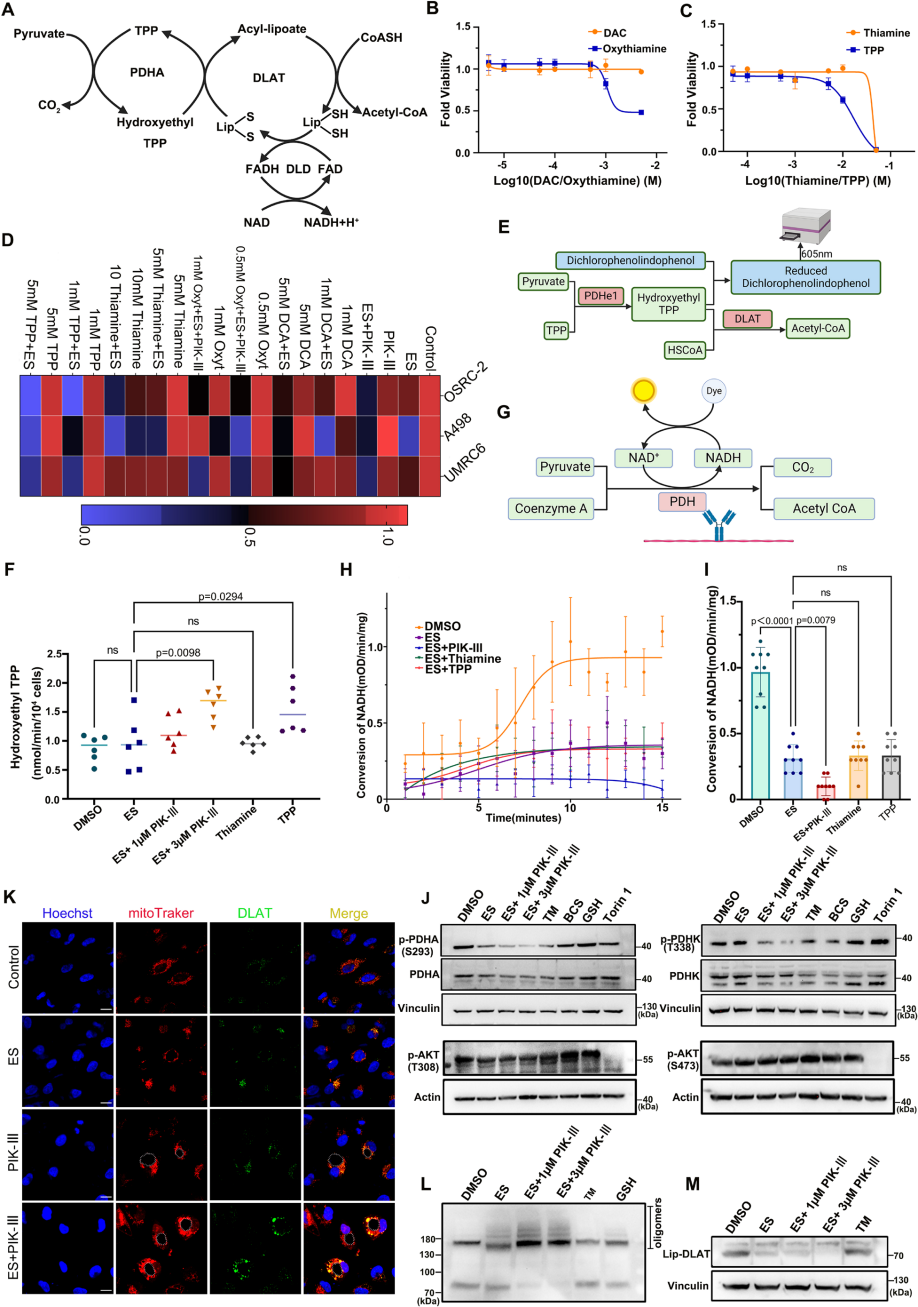
PIK‐III activates PDHA1 through thiamine metabolism and PDHA1 dephosphorylation. (A) Schematic of PDH complex in pyruvate decarboxylation. (B–D) Viability assays of OSRC‐2 cells following co‐culture with various compounds affecting cuproptosis sensitization. (E–F) Hydroxy‐thiamine TPP level assays in OSRC‐2 cells, detecting intracellular hydroxyethyl TPP levels (means± SD, *n* = 6, one‐ way ANOVA). (G–I) PDH Enzyme Activity Microplate assays for PDH complex activity in OSRC‐2 cells post‐lysis. (H) *n* = 3, means ± SD, and (I) *n* = 9, one‐way ANOVA. (J) Phosphorylation levels of PDHA, PDHK and AKT in A498 cells following co‐culture with elesclomol (40 nM) and different concentrations of PIK‐III (1 μM, 3 μM) and TM (20 μM), BCS (20 μM), GSH (10 mM), and Torin1 (10 μM) for 8 h. (K) Immunofluorescence of elesclomol (40 nM) co‐treated with PIK‐III (1 μM) in A498 cells for 8 h. Green, DLAT; red, mitoTraker; blue, Hoechst. Scale bar: 10 μm. (L) Changes in the level of oligomerization of DLAT in OSRC‐2 cells following co‐culture for 8 h with elesclomol (40 nM) and different concentrations of PIK‐III (1 μM, 3 μM) and TM (20 μM) and GSH (10 mM). (M) Comparison of the changes in the levels of lipoylated DLAT in OSRC‐2 cells post 8 h of co‐culture with elesclomol and PIK‐III or two‐drug treatment combined TM (20 μM).

Within the pyruvate dehydrogenase (PDH) complex, each subunit contributes cooperatively to pyruvate decarboxylation. The E1 subunit, PDHA1/PDHB, catalyses the dehydrogenation of pyruvate, while the E2 subunit, DLAT, facilitates the transfer of the hydroxyethyl group to form acetyl‐CoA. Subsequently, the E3 subunit, DLD, transfers the hydrogen to NAD^+^, yielding NADH + H^+^ (Figures 2A and 5A). Given thiamine's role as a precursor for TPP, we investigated whether thiamine or TPP could enhance RCC cells' susceptibility to cuproptosis. To discern whether cuproptosis sensitisation was due to the specific structure of thiamine or the inhibition of PDHK, RCC cells were treated with thiamine, TPP, dichloroacetic acid (DCA), and oxythiamine across a range of concentrations. This treatment resulted in a dose‐dependent decrease in cell viability in OSRC‐2 cells (Figure 5B,C). When these compounds were combined with ES‐Cu, TPP alone demonstrated synergistic potential in enhancing cell viability (Figure 5D).

DCA, a known glycolysis inhibitor, targets PDHK, promoting the dephosphorylation of PDHA1 and redirecting glucose metabolism from anaerobic glycolysis to mitochondrial respiration [32]. Although DCA has been reported to synergise with ES at higher concentrations, its impact on cuproptosis and biosafety in RCC requires further investigation [29, 34]. Our results indicate that PIK‐III treatment leads to intracellular accumulation of hydroxyethyl TPP (Figure 5E,F), and the combination of TPP with ES‐Cu was more effective in elevating hydroxyethyl TPP levels in renal cancer cells than thiamine alone. This suggests that the functional activation of PDHA1, induced by increased thiamine, may counteract the TCA cycle inhibition effect of ES.

Utilising a PDH viability assay, we observed that the intracellular PDH complex precipitates, allowing the E3 component (DLD) to convert NAD^+^ to NADH + H^+^ following the lipoylation of the E2 component (DLAT) (Figure 5G). The addition of PIK‐III resulted in a continued decrease in intracellular NADH production (Figure 5H,I), and the supplementation of thiamine or TPP did not further decrease NADH production based on ES‐Cu treatment. Furthermore, PIK‐III may modulate the phosphorylation level of AKT by targeting PI3K (Figure S5C,D), subsequently affecting the phosphorylation of PDHK (Figure 5J) [35, 36, 37]. A decrease in PDHK phosphorylation attenuated its modification of PDHA1, enhancing PDHA1 activity and promoting pyruvate entry into the aerobic respiratory pathway, resulting in increased hydroxyethyl TPP formation over lactate [29, 38, 39]. Consequently, excessive oligomerisation of the E2 component (DLAT) within the PDH complex may disrupt the metabolic nexus between aerobic respiration and glycolysis (Figures 5A and 8E).

### 3.6 PIK‐III‐Induced Oxidative Stress Enhances Cuproptosis Susceptibility

Intracellular redox homeostasis is pivotal for countering cuproptosis, as an excess of free copper ions is typically sequestered by metallothioneins or glutathione (GSH) [8, 39]. These molecules bind to Cu^2+^ or Cu^+^, thereby inhibiting cuproptosis. The reduction of intracellular GSH levels, achievable through hydrogen peroxide or buthionine sulfoximine (BSO) treatment, has been shown to increase cells' susceptibility to cuproptosis [15, 40, 41]. Our study observed a decrease in both taurine and GSH levels in tumour cells treated with a combination of ES and PIK‐III, indicating the presence of significant oxidative stress (Figures 4A and S5A) [42].

**FIGURE 6:**
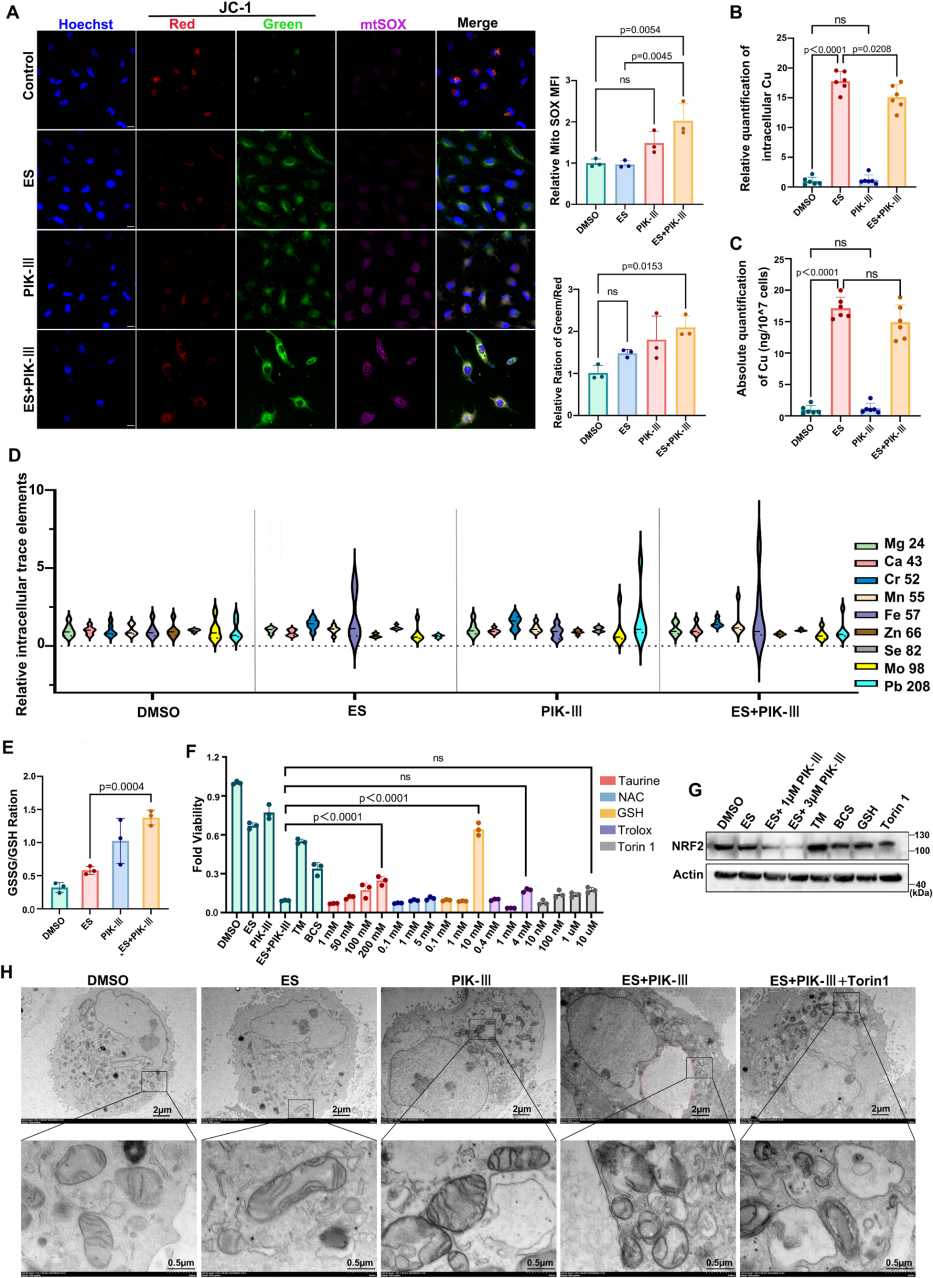
Oxidative stress in PIK‐III‐mediated sensitization of cuproptosis. (A) Mitochondrial membrane potential and the levels of intra‐mitochondrial superoxide in A498 cells were evaluated under treatment with ES‐Cu and PIK‐III (1 μM) for 8 h using JC‐1 and mito‐SOX reagents (*n* = 3, One way ANOVA). White lines mark 10 μm. (B‐C) ICP‐MS detection of intracellular copper content in A498 cells (*n* = 6, ns: Not significant, one‐way ANOVA). (D) Trace element detection in A498 cells via ICP‐MS (*n* = 6, data are means± SD). (E) Ratio of oxidised to reduced glutathione in A498 cells under combined ES‐Cu and PIK‐III treatment for 6 h (*n* = 3, two tailed unpaired t‐test). (F) Cell viability assays of mitigation treatments for PIK‐III induced cuproptosis sensitization using reducing agents of Taurine, GSH, Trolox, and the autophagy agonist Torin1 at different concentrations (*n* = 3, data are means± SD, ns: Not significant, one‐way ANOVA). (G) Immunoblotting for NRF2 protein levels in A498 cells following two‐drug combination treatment of elesclomol (40 nM) and PIK‐III, and simultaneously treated with the copper ion chelator (20 μM TM, 20 μM BCS), or 10 mM reduced glutathione, 10 μM Torin1. (H) Representative Bio‐TEM images of A498 cells after treatment with DMSO, ES‐Cu, PIK‐III (1 μM), ES+PIK‐III and ES+PIK‐III+ Torin1 (Torin1 10 μM). The black box and arrow marked the location of mitochondria. Red dotted line indicated macropinosome.

Immunofluorescence analysis of cells from the dual‐drug treatment group demonstrated accumulation of DLAT around intracellular macropinosomes (Figure 5K), suggesting enhanced oligomerisation upon PIK‐III addition. Western blot analysis substantiated that PIK‐III increased the oligomerisation of DLAT, an effect partially reversible with tetrathiomolybdate (TM) and GSH (Figure 5L). Concurrently, the level of lipoylated DLAT decreased with PIK‐III treatment (Figure 5M). JC‐1 and Mito‐SOX staining of A498 cells further revealed a reduction in mitochondrial membrane potential and an increase in mitochondrial superoxide, indicative of induced oxidative stress by PIK‐III (Figure 6A).

To ascertain changes in total intracellular copper content, Inductively Coupled Plasma‐Mass Spectrometry (ICP‐MS) was utilised. The results showed no increase in intracellular copper levels post‐PIK‐III treatment, but instead a slight decrease was noted (Figure 6B,C). Additionally, no significant differences in other intracellular trace elements were observed among the treatment groups (Figure 6D). GSH levels, measured colorimetrically, were significantly reduced upon PIK‐III treatment (Figure 6E), corroborating the oxidative stress hypothesis.

According to metabolomics data and complementary experiments, the addition of reductive substances and the mTOR inhibitor torin1 to the co‐culture medium of cells treated with ES and PIK‐III attenuated the sensitisation effect of PIK‐III to some extent, with GSH being more effective (Figure 6F). The downregulation of the PI3K‐AKT pathway and the significant decrease in NRF2 protein levels in the ES+PIK‐III group suggest that PIK‐III potentially regulates intracellular GSH levels via the PI3K‐AKT‐NRF2 signalling pathway, a mechanism previously reported [43]. Transmission electron microscopy (TEM) of A498 cells exposed to PIK‐III, with or without ES, provided further evidence of induced oxidative stress, characterised by mitochondrial membrane thickening and swelling (Figure 6H). Moreover, TEM analysis confirmed that the vacuoles, previously identified as macropinosomes, are single‐membrane structures and are not associated with autophagic lysosomes (Figure 6H).

In summary, PIK‐III‐induced oxidative stress, evidenced by decreased NRF2 and GSH levels, is a significant contributor to the sensitisation of cuproptosis. This finding also rationalises the observed reduction in FDX1 and LIAS expression following PIK‐III intervention (Figure 2I).

### 3.7 Synergistic Antitumor Efficacy of PIK‐III and ES In Vivo

**FIGURE 7:**
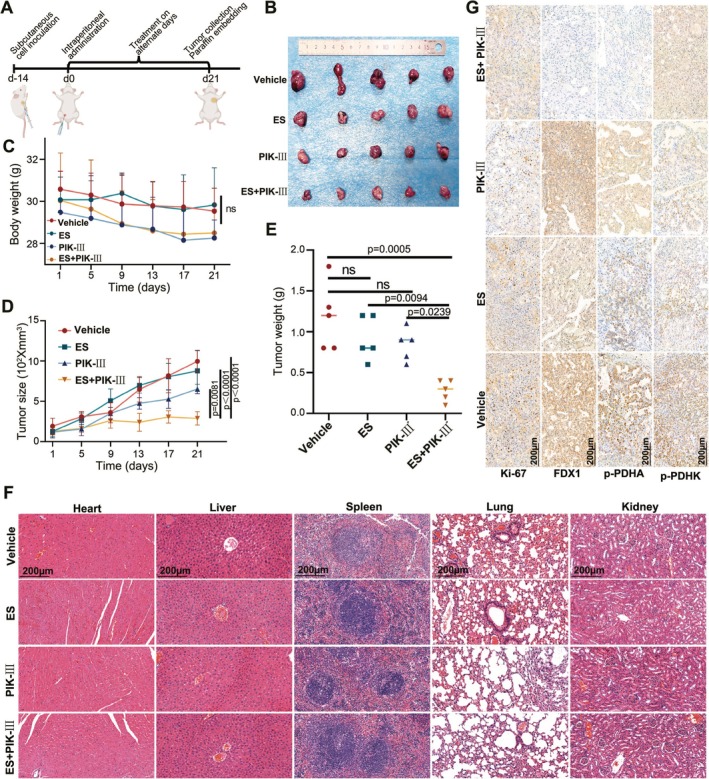
In vivo validation of PIK‐III Sensitisation to elesclomol‐induced cuproptosis. (A, B) BALB/C nude mice were treated with intraperitoneal injections of equal volumes of drug solvents, elesclomol (20 mg/kg), PIK‐III (10 mg/kg), and two‐drug combination (elesclomol 20 mg/kg + PIK‐III 10 mg/kg) for 21 days and treated every other day to observe the effects on subcutaneous tumour growth. (C) Statistical analysis of weight changes of nude mice, (D) weight and (E) volume of tumour during treatment, *n* = 5, data are mean ± SD, and *P*‐value was calculated by two‐factor ANOVA (C) or one‐way ANOVA (D, E). (F) Histological evaluation of organ damage post‐treatment via HE staining. (G) Subcutaneous renal cancer cell tumours were stained immunohistochemically after treatment, and markers such as Ki67, cuproptosis‐associated FDX1 and phosphorylated PDHA1 and PDHK were detected.

To assess the in vivo tumour inhibitory effects and biosafety of PIK‐III and ES, we established subcutaneous tumours in nude mice using OSRC‐2 cells. Following confirmation of successful tumour formation, treatment was initiated with intraperitoneal injections of ES. The control group received an equivalent volume of PBS containing DMSO. Treatment groups were as follows: the ES group received 20 mg/kg/day of ES; the PIK‐III group received 10 mg/kg/day; and the combination group received both ES 20 mg/kg/day and PIK‐III 10 mg/kg/day (Figure 7A,B). Injections were administered every other day, with no significant changes in body weight observed across all groups (Figure 7C).

After a 21‐day treatment period, we observed that the tumour volume and weight in the combination therapy group (ES+PIK‐III) were significantly reduced compared to the monotherapy groups, with mean values as follows: Vehicle 997.3 mm^3^, ES 878 mm^3^, PIK‐III 652.9 mm^3^, and ES+PIK‐III 287.8 mm^3^ for volume; and Vehicle 1.18 g, ES 0.92 g, PIK‐III 0.84 g, and ES+PIK‐III 0.28 g for weight (Figure 7D,E). The synergistic antitumour effect of the combination treatment was evident, as the single‐agent groups exhibited only modest tumour growth inhibition.

Notably, no overt organ damage was observed in histological assessments of the heart, liver, spleen, lungs, and kidneys among the treated mice (Figure 7F). Immunohistochemical analysis of the subcutaneous tumours indicated a significant reduction in Ki‐67 expression in the ES+PIK‐III group, suggesting a decreased rate of cellular proliferation. Additionally, a decrease in FDX1 expression and a trend towards decreased phosphorylation of PDHA1 and PDHK were observed in the same group, indicating potential disruptions in cuproptosis‐related pathways (Figure 7G). Continuous administration of PIK‐III significantly inhibited tumour growth, and the combination of ES with PIK‐III at the administered doses did not result in detectable harm to vital organs.

### 3.8 Synergy Effect of PIK‐III and ES in Patient‐Derived Tumour Slice Model and PDX

**FIGURE 8:**
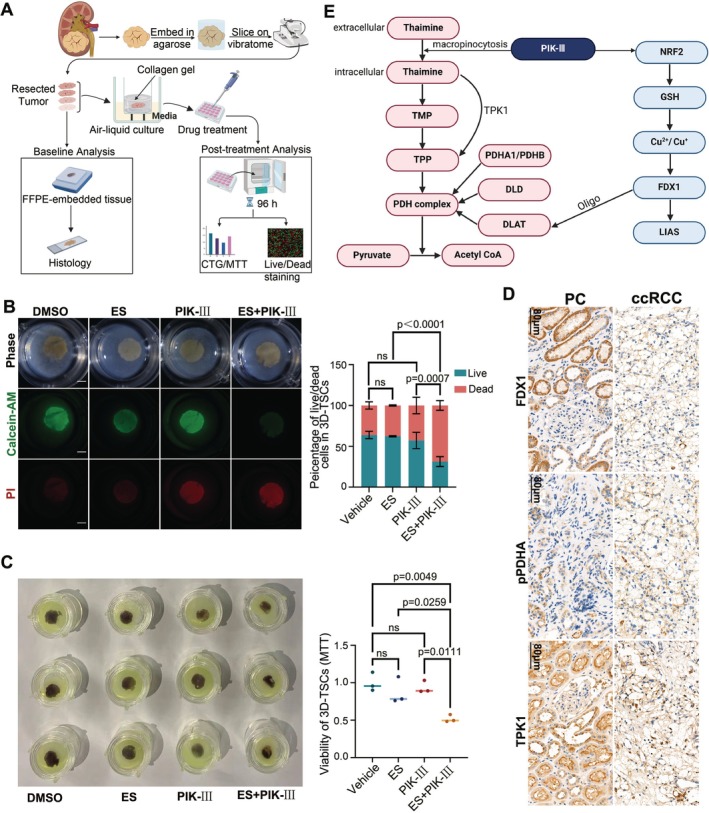
Validation of PIK‐III sensitization of elesclomol‐induced cuproptosis by tumour tissue section culture model. (A) Schematic of drug induced tumour killing in clinical samples. Tumour was co‐cultured by drug‐containing medium through a clinical renal cancer tissue section culture system. (B) Live‐dead cell staining of renal cancer tissues after 96 h of drug treatment was performed by PI and Calcein‐AM dyes. Scale bar: White line 1 mm. Proportions of live/dead cells in renal cancer tissue section after different treatments (*n* = 3, two‐way ANOVA). (C) Tumour tissue slices were subjected to MTT cell activity assay after drug treatment (*n* = 3, One‐way ANOVA). (D) Immunohistochemical comparison of renal carcinoma (ccRCC) and paracancerous (PC) tissues from representative patient with renal clear cell carcinoma. (E) Summary diagram depicting the intracellular molecular mechanism by which PIK‐III mediates cuproptosis sensitization.

The 3D organoid culture system is recognised for its ability to preserve the tumour microenvironment and accurately mimic the biological characteristics of human tumours [44]. Utilising human renal cancer tissues, we generated patient‐derived tumour slices (PDTS) to assess the efficacy of various treatments (Figure 8A). Following a 4‐day culture period with drug‐containing medium, which was refreshed at 48‐h intervals, live‐dead cell staining was conducted. This analysis demonstrated a significant reduction in Calcein‐AM fluorescence intensity in tissue slices treated with the combination of drugs, indicative of diminished cell viability (Figure 8B). Specifically, the Calcein‐AM/PI% values were as follows: DMSO 63.87%, ES 62.36%, PIK‐III 57.19%, and ES+PIK‐III 31.25%.

Concurrently, there was a marked increase in propidium iodide (PI) fluorescence within the tissue sections, suggesting that the dual‐drug treatment exerted a synergistic cytotoxic effect on the renal cancer cells. An MTT assay, conducted after removing the hydrogel, corroborated these findings, showing a significant decrease in cell viability in the combination treatment group compared to the single‐agent groups (Figure 8C). The average relative cell viability was 87.57% for ES, 93.71% for PIK‐III, and 51.82% for the ES+PIK‐III combination. These results substantiate the synergistic antitumour activity of the two‐drug combination, as the viability inhibition in the single‐agent groups was less than 20% of that in the control group. We also established Patient‐Derived Xenograft (PDX) models using fresh tumour tissues obtained from clinical kidney cancer patients (Figure S8A). Subcutaneous implantation of renal cancer tissues was performed in 6‐week‐old NSG mice. Drug treatment began the following day (Day 1), and mice were euthanised 35 days after the initiation of treatment. The inhibitory effects of ES combined with PIK‐III on tumour growth were evident in both the volume and final weight of the PDX tumours (Figure S8B–D). These results suggest that PIK‐III effectively enhances the antitumour efficacy of ES in the treatment of renal cancer in the PDX model.

Further analysis using immunohistochemistry on human renal clear cell carcinoma tissue sections indicated significantly reduced expression of FDX1 and TPK1 in tumour tissues compared to paracancerous tissues (Figure 8D). The downregulation of TPK1 may be associated with the tumour cells' impaired capacity to convert thiamine into its active form, TPP, which is essential for the pyruvate dehydrogenase (PDH) complex's function (Figure 4B,D). Consequently, a reduction in TPP levels is expected to compromise the PDH complex's activity, further implicating thiamine metabolism in the response to cuproptosis‐inducing therapies.

## 4 Discussion

Hypoxia and aerobic glycolysis are hallmark metabolic features of tumour cells; however, effective clinical strategies to modulate these states remain elusive [16, 44]. While these conditions are closely linked to cuproptosis, no studies have reported on altering tumour glucose metabolism to influence cuproptosis.

In this study, we identified PIK‐III as the most potent sensitiser of cuproptosis from a library of molecular inhibitors targeting aerobic glycolysis. PIK‐III demonstrated significant synergy with ES, even under hypoxic conditions typically associated with cuproptosis resistance. Employing multi‐omics techniques, we explored the mechanisms behind PIK‐III's sensitisation effects, hypothesising a regulatory role in cuproptosis related to thiamine metabolism, macropinocytosis, and oxidative stress. The synergistic antitumour effect of PIK‐III and ES was further validated in animal models and patient‐derived tumour slices (PDTS), with safety profiles supporting potential clinical translation.

PIK‐III, an orally active inhibitor of vacuolar protein sorting 34 homologue (VPS34), targets both PI3K and mTOR pathways and has been utilised as an autophagy inhibitory tool [36, 37]. VPS34's role in cellular membranes and dynamic transport of substances, through the conversion of phosphatidylinositol to phosphatidylinositol‐3‐phosphate, modulates endocytosis transport [36, 37, 45]. While regulating autophagy, the VPS34 protein is involved in the regulation of the nuclear EGFR signalling pathway, and hexokinase 2 (HK2)‐dependent glycolysis was also down‐regulated which is one of the bases for PIK‐III acts as an aerobic glycolysis inhibitor [46, 47]. Due to the effect of PIK‐III on membrane ruffles, it is hypothesized that its pharmacological treatment also severely impairs the uptake of dextran during the formation of macropinosome [48]. In the presence of PIK‐III, macrophages exhibit an absence of membrane vesicle formation, and their capacity to internalise fluorescent liquid phase tracers is significantly compromised [48]. Consequently, PIK‐III elicits distinct phenotypic responses in endocytosis and mitochondrial respiration across different cell types. Notably, in this study, PIK‐III treatment was observed to diminish glycolytic activity in renal carcinoma cells, thereby rendering them more susceptible to cuproptosis, a finding that suggests its potential as a therapeutic agent for enhancing cuproptosis sensitivity.

PIK‐III's induction of macropinocytosis and facilitation of thiamine uptake by renal cancer cells offer a comprehensive strategy to inhibit glucose metabolism in both anaerobic and aerobic respiration pathways. Our experiments demonstrate that PIK‐III promotes thiamine metabolism and activates PDHA1, increasing pyruvate entry into the TCA cycle. Concurrently, ES‐induced DLAT oligomerization obstructs the TCA cycle, reducing intracellular glucose metabolism in RCC. This leads to decreased lactate production and increased hydroxyethyl TPP levels. The low expression of thiamine metabolism‐related channels in RCC suggests a ‘thiamine‐depleted’ state, which macropinocytosis may alleviate by facilitating thiamine uptake for aerobic respiration (Figure 8E,F). This led to decreased intracellular lactate production and elevated hydroxyethyl TPP levels (Figures 5F and S3F). Since cells can only produce TPP by acquiring extracellular thiamine, incremental hydroxyethyl TPP may exhaust thiamine pool [31, 33, 49, 50]. The low expression of thiamine metabolism‐related channels and their association with the prognosis of RCC patients strongly suggest a ‘thiamine‐depleted’ state in RCC (Figure 4C,D) [31, 32]. Macropinocytosis is hypothesized to serve as a nutrient acquisition mechanism for tumour cells under conditions of nutrient scarcity [33]. In the context of renal cancer cells, where the uptake of thiamine and TPP via thiamine transporter 1 (THTR1) and thiamine pyrophosphokinase (TPPT) is insufficient, PIK‐III‐induced macropinocytosis emerges as a compensatory mechanism to facilitate thiamine acquisition for aerobic respiration. During cuproptosis, the oligomerization of dihydrolipoamide S‐acetyltransferase (DLAT) impedes the timely dehydroxylation of hydroxyethyl thiamine pyrophosphate (hydroxyethyl TPP), leading to TPP deficiency and the subsequent blockade of pyruvate decarboxylation (Figure 5A). The macropinocytosis‐mediated thiamine uptake in RCC mitigates TPP deficiency, promotes pyruvate decarboxylation, and potentially reduces the reliance on the Warburg effect. Conversely, the excessive fusion of lysosomes and endosomes, a consequence of macropinocytosis, may impair lysosomal protein degradation [45, 51]. Accumulation of oligomerized DLAT and dysfunctional mitochondria, due to the lysosomal overload and diminished degradation capacity, has been observed around these organelles (Figures 5K and 6H).

Previous research has highlighted the potential of thiamine metabolism in enhancing the viability of the pyruvate dehydrogenase (PDH) complex; however, the underlying mechanisms have not been fully elucidated [49, 51]. Thiamine has demonstrated antitumour properties at high doses, potentially through inhibiting pyruvate dehydrogenase kinase (PDHK), which results in reduced phosphorylation of the E1 subunit of PDH (PDHA1) (Figure 5C,D,J) [31, 45, 49]. Despite these findings, the limited bioavailability of thiamine may restrict its clinical efficacy in cancer therapy. Some studies have indicated that TPP, the active form, is responsible for inhibiting tumour cell proliferation in vitro [31].

In this study, we observed that treatment with PIK‐III significantly increased intracellular thiamine levels in RCC and concurrently elevated levels of thiamine‐related derivatives, such as thiamine monophosphate (TMP) and TPP (Figures 4A and S5A,B). Copper ions (Cu^2+^) have been implicated in mediating cancer cell death, with this process linked to the reduced viability of PDH and α‐ketoglutarate dehydrogenase (α‐KGDH) [45]. Notably, the presence of thiamine during co‐culture experiments has been shown to significantly reduce copper‐ion‐associated tumour cell death [45], a finding that appears to contrast with our observations. However, the concept of cuproptosis suggests that the dysfunction of the TCA cycle induced by ES is due to DLAT oligomerization, which impairs the activity of PDH and α‐KGDH complexes [15]. Our experiments confirm that PIK‐III‐mediated increases in intracellular thiamine levels or the dephosphorylation of PDHA1 can enhance the sensitivity of renal cancer cells to ES (Figures 4E,F and S5D,J). These findings indicate that the initiation of cuproptosis is specifically associated with the E2 component (DLAT) within the PDH complex and does not involve the loss of the entire complex's activity (Figure 5A,G–I). Importantly, elevating intracellular thiamine or TPP levels can enhance the capacity of PDHA1 for pyruvate decarboxylation. The impact of PDHA1 on cellular reliance on aerobic respiration remains independent of the degree of DLAT oligomerization or lipoylation, despite the ongoing proteotoxicity and functional inhibition caused by DLAT oligomerization [15].

Based on these observations, we propose that PIK‐III promotes endosome formation via macropinocytosis, thereby facilitating the uptake of extracellular thiamine in renal cancer cells to mitigate ‘thiamine‐depleted’ state, particularly since both THTR1 and TPPT are under‐expressed in kidney renal clear cell carcinoma (KIRC) (Figure 4C). The protein levels of thiamine metabolism‐related channels were not significantly altered; however, intracellular thiamine levels decreased with the addition of EIPA (Figure 4E–G). In conclusion, elevating intracellular thiamine and its derivatives primarily enhances pyruvate decarboxylation at the entry point of the TCA cycle, but does not counteract the TCA cycle inhibition induced by ES, nor does it impede the onset of cuproptosis.

## Author Contributions

Y.D., Z.Z., and J.W. provided overall supervision of the project. Z.Z., D.X., and W.C. were responsible for the experimental design. In vivo experiments were conducted by D.X., while D.X. and Y.W. carried out in vitro studies. Experiments involving 3D TSCs were performed by D.X., M.Y., and X.W. Data analysis and manuscript preparation were undertaken by D.X. and X.W. All authors contributed to the final approval of the submitted manuscript.

## Disclosure

All the experiments are representative of at least three independent repeats. Data are shown as mean ± SD. *p* values were determined using the following statistical tests as indicated in figure legends: Two‐tailed Student's *t*‐test, with a 95% confidence threshold; *p* < 0.05 was determined as the statistical significance threshold. One‐way or Two‐way ANOVA (Fisher's LSD) (*α* < 0.05); Exact test *p*‐value (FDR) (*α* < 0.05). GraphPad Prism 9.5.1 was used for data presentation, graphing and statistical analyses.

## Ethics Statement

This study was approved by the Research Ethics Committee of The Fifth Affiliated Hospital of Sun Yat‐sen University (approval number: K30‐1‐2022), and informed consent was obtained from each patient. The animal procedures were conducted according to the standards of the National Institutes of Health Guide for the Care and Use of Laboratory Animals and approved by the Animal Ethics Committee of The Fifth Affiliated Hospital of Sun Yat‐sen University (2022061504).

## Conflicts of Interest

The authors declare no conflicts of interest.

## Supporting information


Data S1.

## Data Availability

The data that support the findings of this study are available from the corresponding author upon reasonable request.
