# Supplementary material for: PIK‐III‐Mediated Elevation of Thiamine Re‐Sensitises Renal Cell Carcinoma to Cuproptosis via Activating PDHA1
Source: Cell Prolif. 2025 Jul 31;59(3):e70101. doi: 10.1111/cpr.70101 (PMC12961540; doi:10.1111/cpr.70101)
Supplement: Supplementary file 1 — Data S1. [file CPR-59-e70101-s001.docx]

**Supporting Information for**

PIK-III-mediated elevation of thiamine re-sensitizes renal cell carcinoma to cuproptosis via activating PDHA1

**Runing Title:** PIK-III sensitizes cuproptosis in ccRCC

Dongdong Xie^1,2^, Yu Wang^1,2^, Wenjie Cheng^1^, Minbo Yan^1^, Kunyu Li^2,4^, Xiang Wu^1,2^, Jiaqing Wu^3,*^, Zhuangzhuang Zhang^2,4*^ and Yingbo Dai^1,2,*^

^1^Department of Urology, The Fifth Affiliated Hospital of Sun Yat-sen University, China.

^2^Guangdong Provincial Engineering Research Center of Molecular Imaging, the Fifth Affiliated Hospital, Sun Yat-sen University, Zhuhai 519000, Guangdong, China

^3^Department of Kidney Transplantation, The Third Affiliated Hospital of Sun Yat-sen University, China.

^4^Guangdong-Hong Kong-Macao University Joint Laboratory of Interventional Medicine, the Fifth Affiliated Hospital, Sun Yat-sen University, Zhuhai 519000, China.

**^*^Correspondence:** Yingbo Dai, daiyingbo@126.com, Department of Urology, The Fifth Affiliated Hospital of Sun Yat-Sen University, No. 52, Meihua East Road, Zhuhai, 519000, Guangdong, China. Zhuangzhuang Zhang, [zhangzhzh36@mail.sysu.edu.cn](mailto:zhangzhzh36@mail.sysu.edu.cn), Guangdong Provincial Engineering Research Center of Molecular Imaging, The Fifth Affiliated Hospital of Sun Yat-Sen University, No. 52, Meihua East Road, Zhuhai, 519000, Guangdong, China. Jiaqing Wu, [wujq35@mail.sysu.edu.cn](mailto:wujq35@mail.sysu.edu.cn). Department of Kidney Transplantation, The Third Affiliated Hospital of Sun Yat-sen University, No.2693, Xuejie Avenue, Guangzhou, 510630, Guangdong, China;

Dongdong. Xie, Yu.Wang and Wenjie. Cheng contributed equally to this work**.**

**This file includes:**Figures S1-8**,** Tables S1 and SI Methods.

**Figures S1-8**

**
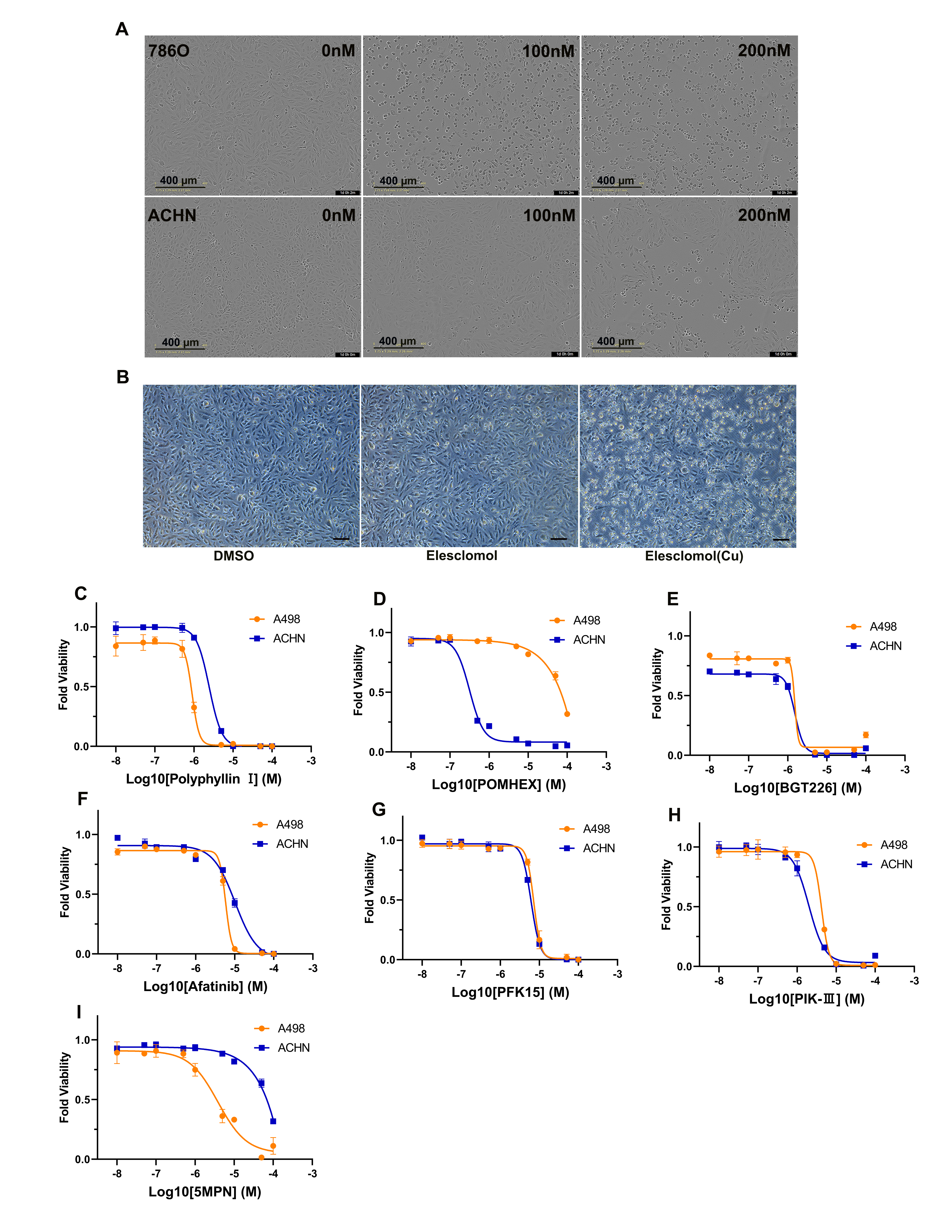
**

**Figure S1** **Cell viability assay in different cell lines.** (**A)** Incucyte was used to observe the changes in survival and proliferation of 786O and ACHN cells under the intervention of different concentrations of elesclomol. (**B)** A498 renal cancer cells treated with 100 nM elesclomol alone and addition of 1 μM copper ions, scale bar: black line marks 100 μm. (**C-I)** Effect of single-agent treatment with screened aerobic glycolysis inhibitors on the viability of different renal cancer cell lines under hypoxic conditions.


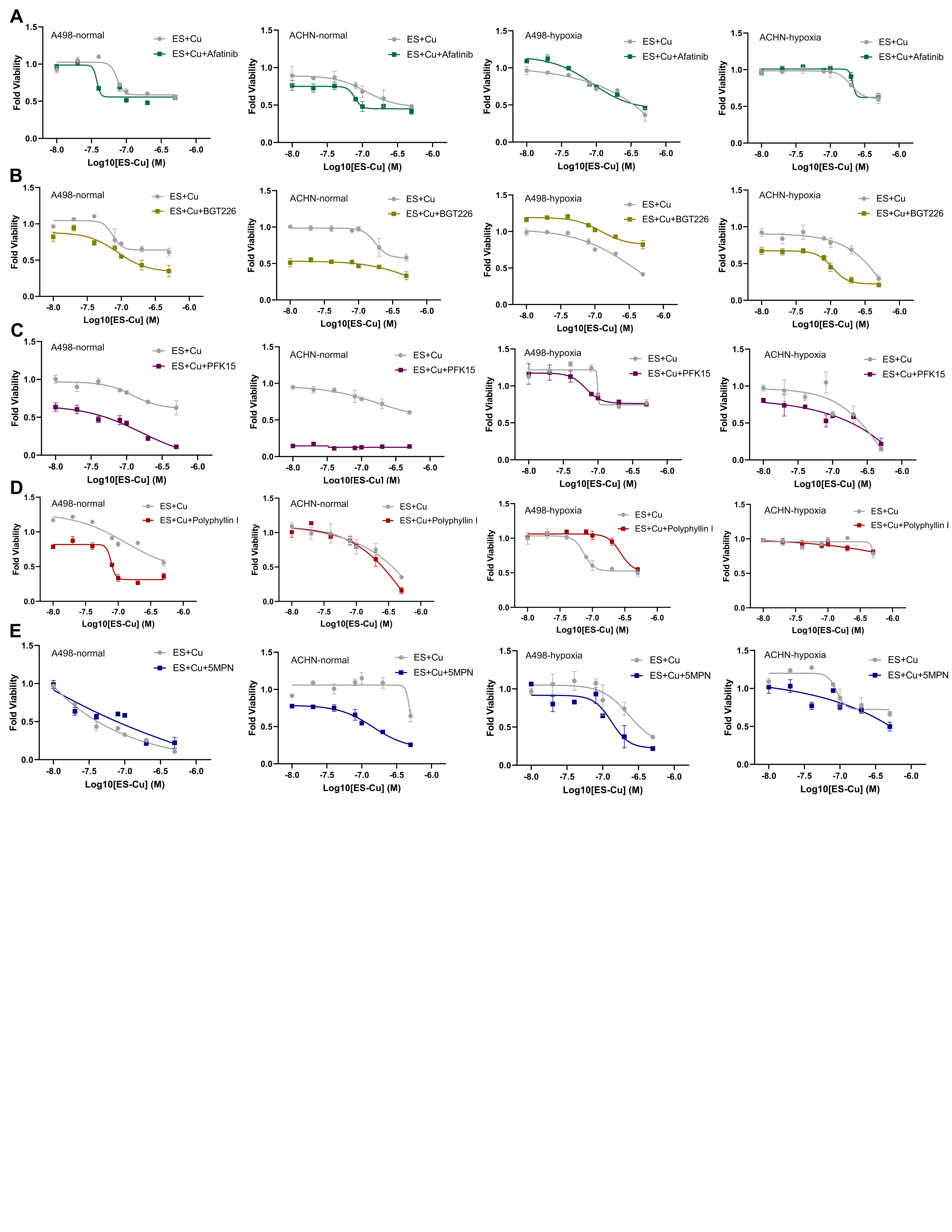


**Fig. S2** **Effect of different glycolysis inhibitors combined with elesclomol at their respective IC_10_ concentrations on the viability of A498 or ACHN cells under normoxic (21% O_2_) or hypoxic (94% N_2_, 5% CO_2_, 1% O_2_) conditions.** **(A)** A498 cells under normoxic O2 and 4 μM Afatinib, ACHN cells under normal oxygen and 4 μM Afatinib, A498 cells under hypoxia and 4 μM Afatinib, ACHN cells under hypoxia and 4 μM Afatinib; **(B)** A498 cells under normal oxygen and 900 nM BGT226, ACHN cells under normal oxygen and 500 nM BGT226, A498 cells under hypoxia and 900 nM BGT226, ACHN cells under hypoxia and 500 nM BGT226, **(C)** A498 cells under normal oxygen and 5 μM PFK15, ACHN cells under normal oxygen and 4 μM PFK15, A498 cells under hypoxia and 5 μM PFK15, ACHN cells under hypoxia and 4 μM PFK15; **(D)** A498 cells under normal oxygen and 0.5 μM Polyphyllin I, ACHN cells under normal oxygen and 1 μM Polyphyllin I, A498 cells under hypoxia and 0.5 μM Polyphyllin I, ACHN cells under hypoxia and 1 μM Polyphyllin I; **(E)** A498 cells under normal oxygen and 1 μM 5MPN, ACHN cells under normal oxygen and 1 μM 5MPN, A498 cells under hypoxia and 1 μM 5MPN, ACHN cells under hypoxia and 1 μM 5MPN. (n= 3, data are mean± SD).


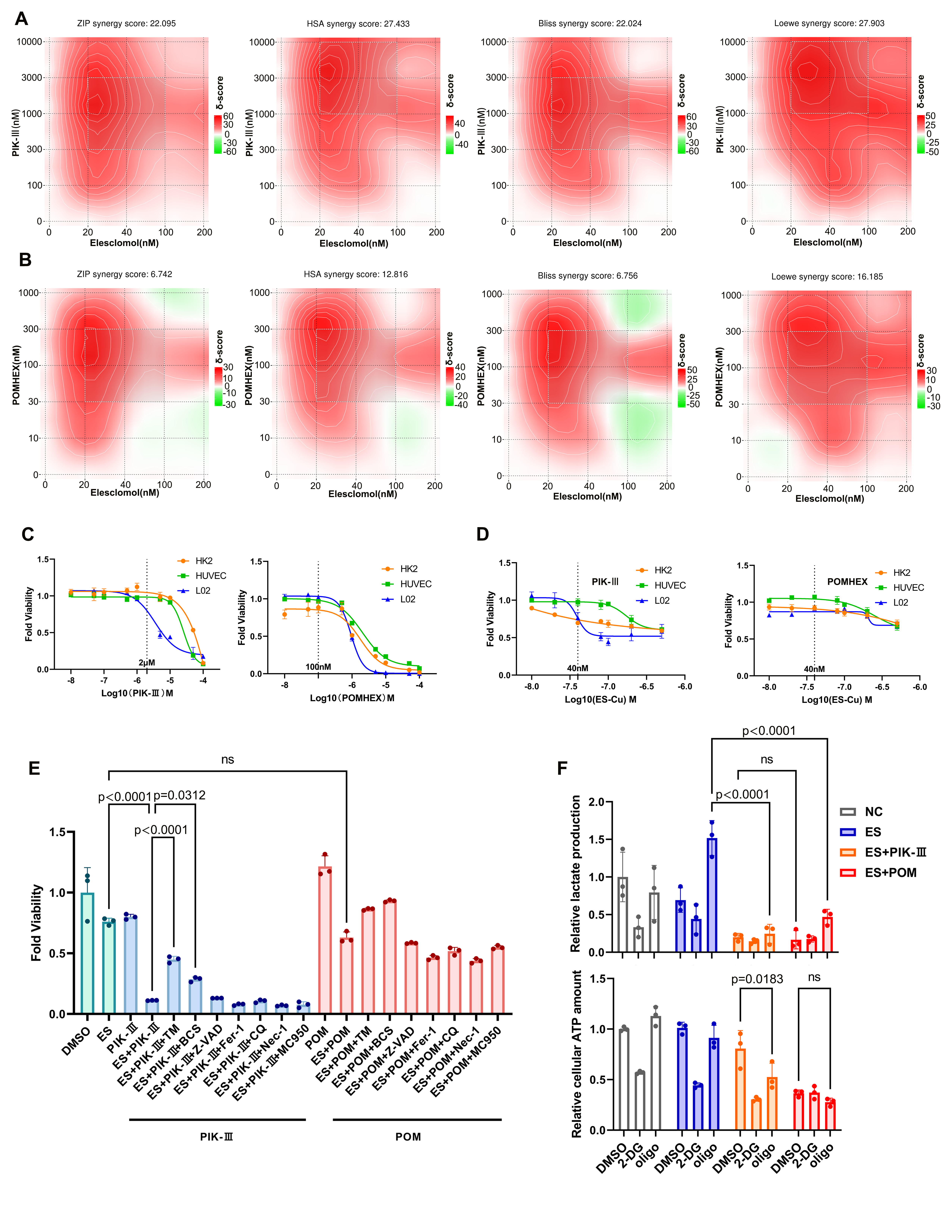


Fig. S3 Cell viability assay in various cell lines with indicated drug treatment. (A-B) The effect of PIK-III/POMHEX on the cell viability of A498 cells using the synergy finder model to assess the drug synergism between glycolysis inhibitors and ES-Cu. Data were analyzed using the synergy finder assessment platform. (C) Viability of different concentrations of PIK-III or POMHEX alone on cell viability in normal cell lines like HK2, HUVEC and L02 (n= 3, data are means± SD). (D) Viability of PIK-III (2 μM) or POMHEX (200 nM) combined with different concentrations of elesclomol in normal cell lines. (E) The effects of PIK-III and POMHEX were assessed by the use of different copper ion chelating agents (TM, 20 μM; BCS. 20μM) and cell death inhibitors like Z-VAD (20 μM), Fer-1 (10 μM), CQ (10 μM), Nec-1 (20 μM), and MC950 (50 nM) were used to assess the mitigation of cuproptosis sensitization by PIK-III or POMHEX combined with elesclomol+ Cu2+ (n= 3, data are means± SD, ns not significant, one-way ANOVA). (F) The effect of Glycolysis/OXPHOS assay reagent on lactate and ATP production in OSRC-2 cells when combining PIK-III or POMHEX (6 hours) and when intervening with 25 mM 2-DG (4 hours) was utilized to assess glycolysis levels, and when intervening with 1.25 μM oligomycin (4 hours) to assess oxidative phosphorylation levels (n= 3, data are means± SD, two-way ANOVA).


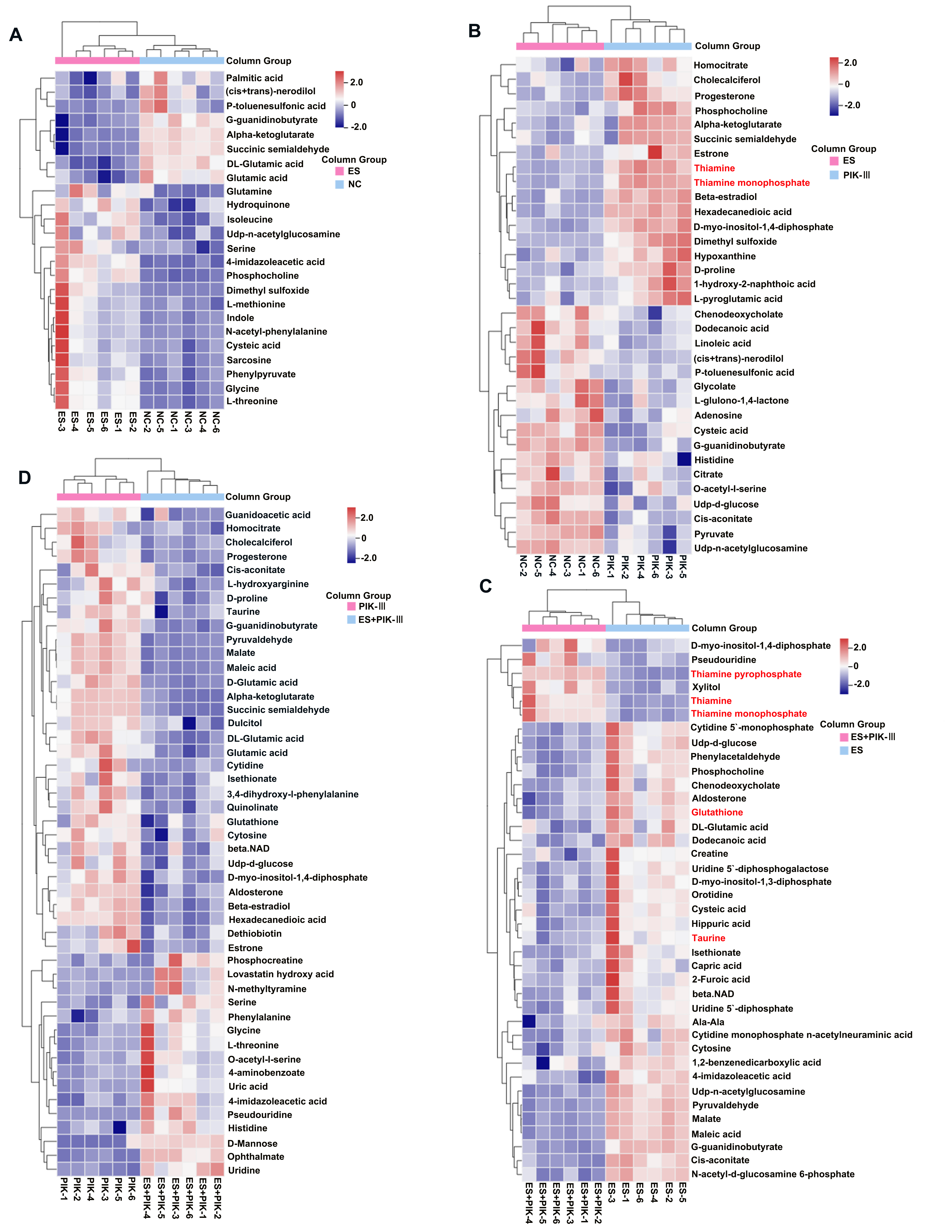


Fig. S4 Heat map of differential intracellular metabolites in A498 cells detected by LC-MS. (A) ES vs NC. (B) PIK-III vs NC. (C) ES+ PIK-III vs ES. (D) ES+ PIK-III vs PIK-III.


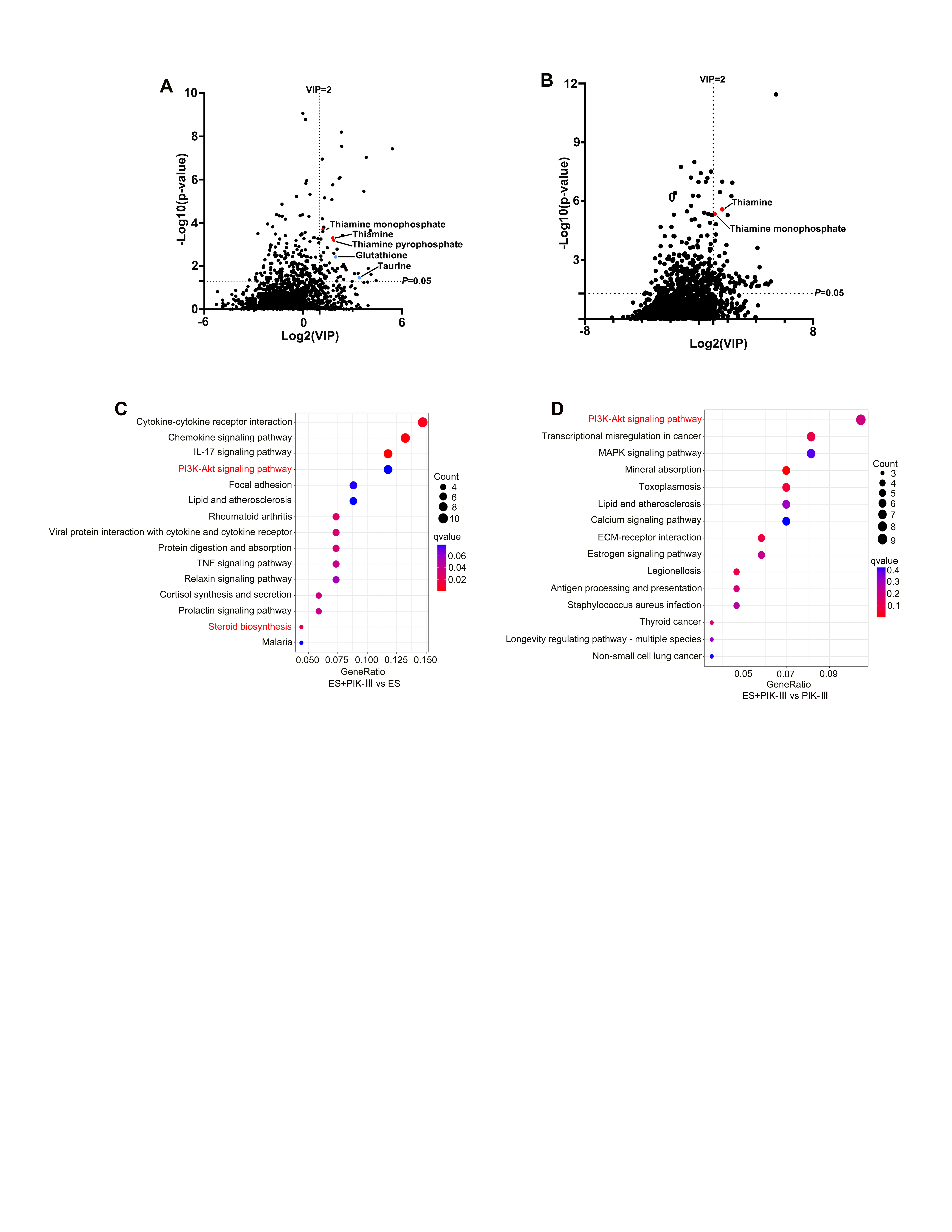


Fig. S5 Untargeted metabolomics analysis to identify potential metabolites. (A-B) Untargeted metabolomics of ES+ PIK-III vs. ES (A) and PIK-III vs NC (B) group both showed changes in thiamine-related metabolites (differential metabolites for VIP> 2, *P*-value< 0.05). (C-D) Transcriptome sequencing KEGG enrichment analysis yielded the down-regulated pathways that were differentially enriched for the genes between each two groups.


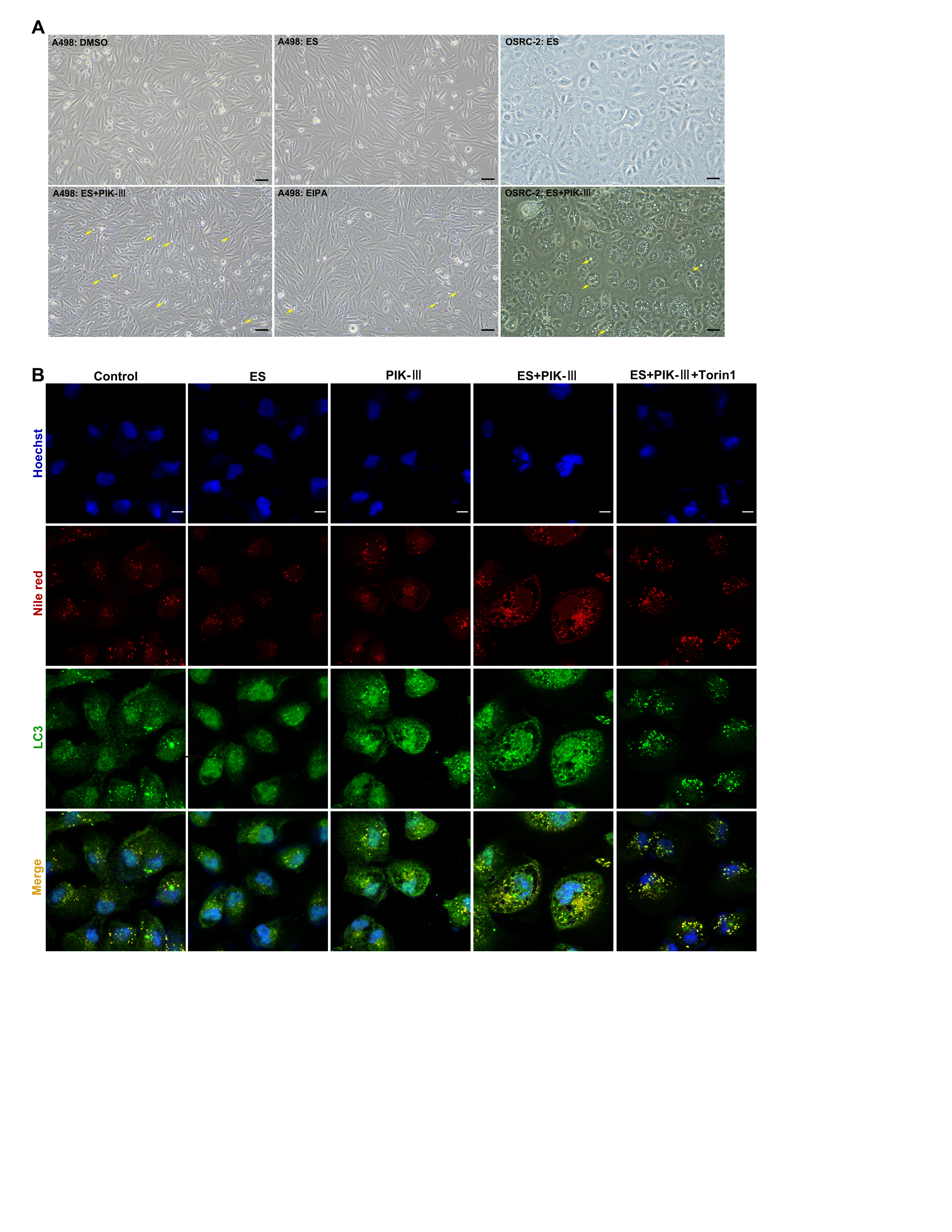


Fig. S6 Morphological changes determined by different methods. (A) Morphology of renal cancer cells after treatment with elesclomol (40 nM), PIK-III (1 μM) and ES+ PIK-III+ EIPA (10 μM) for 6 hours under 40× magnification. Scale bar: black line mark 100 μm, and vacuole-like structures are yellow marked. (B) Nile red staining/LC3 immunofluorescence of OSRC-2 cells co-cultured with elesclomol (40 nM), PIK-III (1 μM), ES+ PIK-III and Torin1 (10 μM) for 8 hours respectively. Scale bar: white line mark 10 μm.


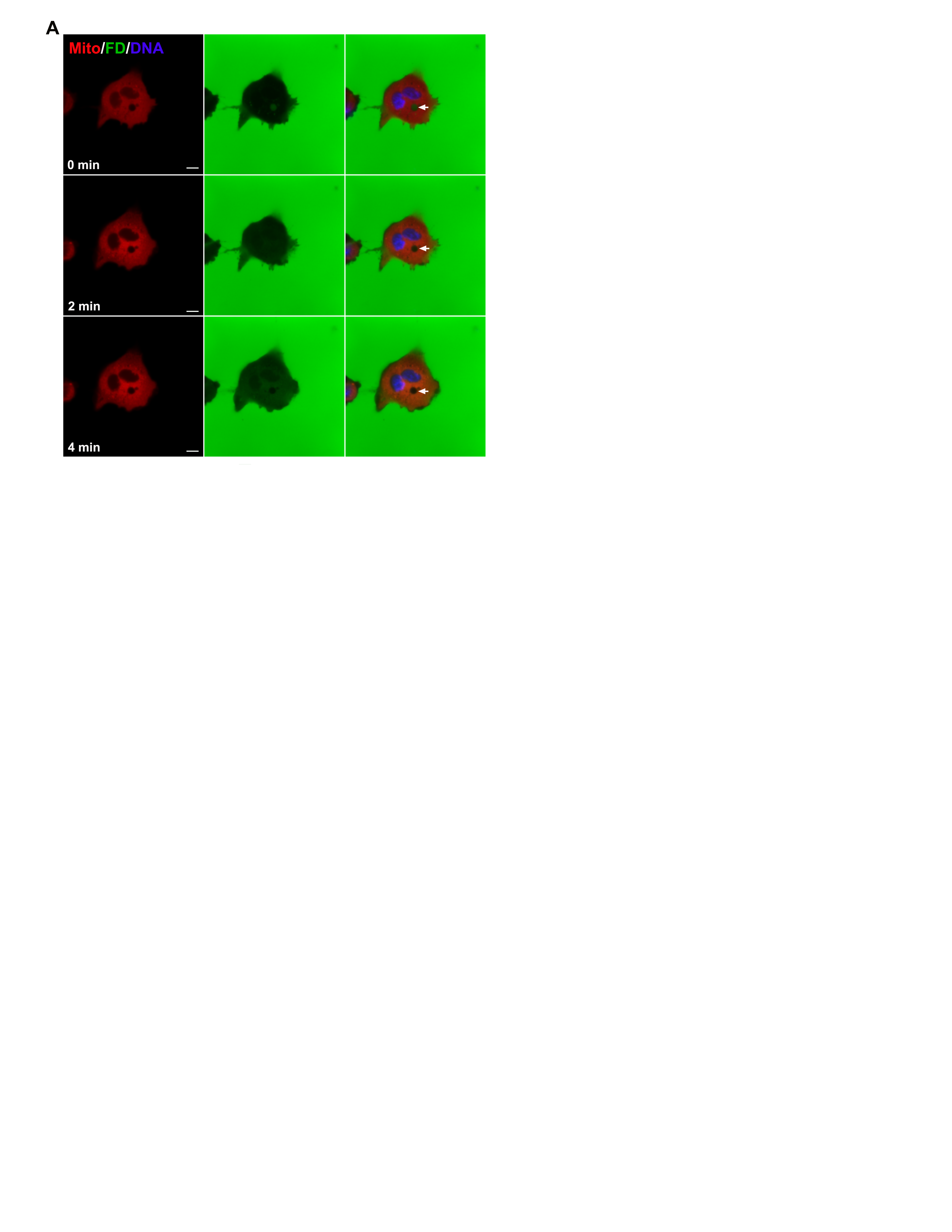


**Fig. S7** Time-lapse images of macropinocytosis and mitochondrial localization in A498 cells after treatment of ES-Cu and PIK-III. Macropinosomes are marked by white arrows. Scale bar = 5 μm.


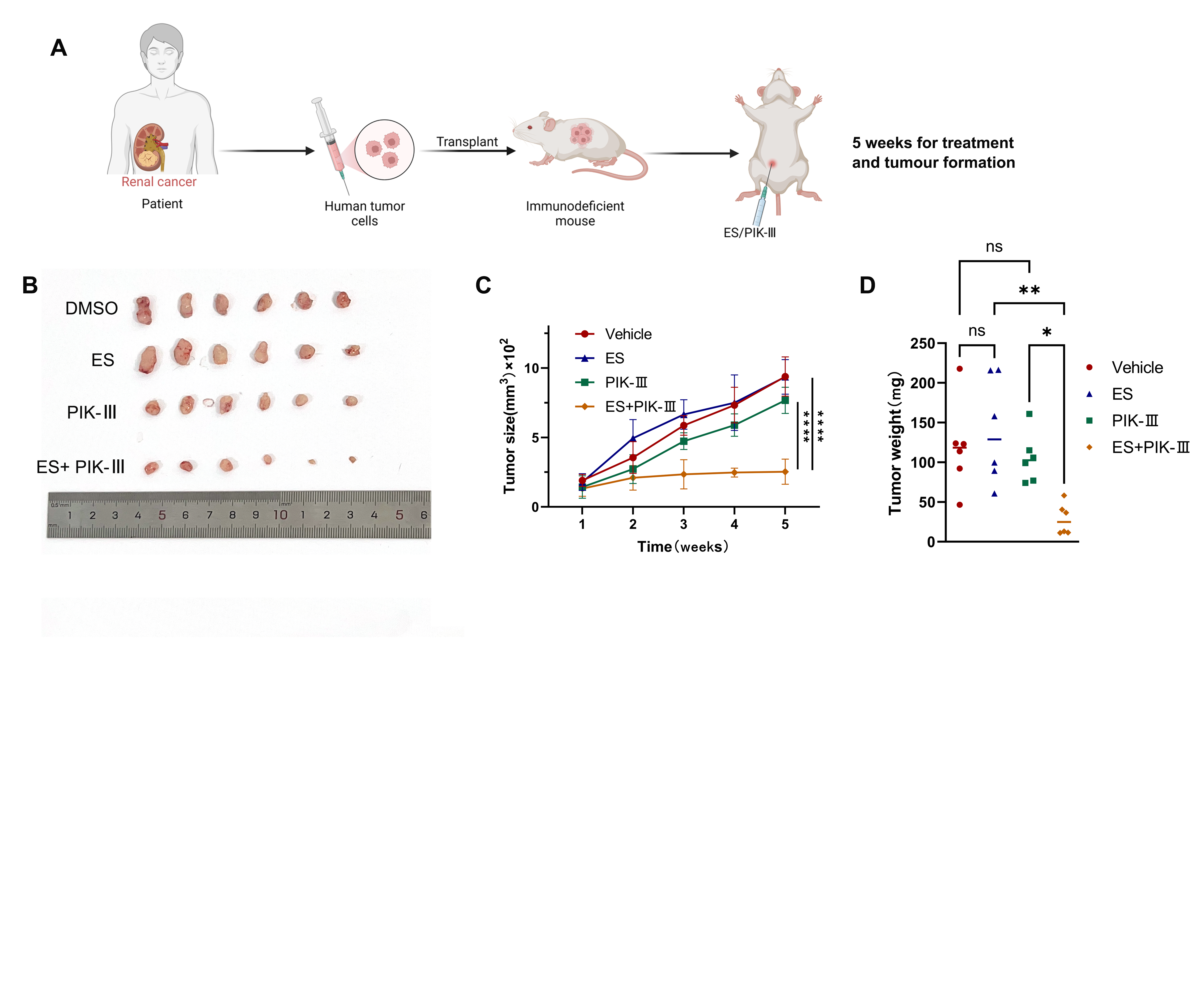


Fig. S8 Establishment of human renal cancer xenograft model in immunodeficient mice and therapeutic efficacy of ES+ PIK-Ⅲ treatments. (A) Schematic diagram of PDX model construction. (B) Representative tumors collected from mice in each treatment group (the dose administered was the same as in the subcutaneous tumour model). (C-D) Analysis of tumor volume progression and final tumor mass in PDX models (n= 6, data are means± SD, One-way ANOVA).

**Table S1 Predicted effective Inhibitory Concentrations of each aerobic glycolysis inhibitor alone in A498 and ACHN cells under hypoxic conditions**

| **Cell line** | **Compound** | **IC_50_ (μM)** | **IC_20_(μM)** | **IC_10_(μM)** |
| --- | --- | --- | --- | --- |
| **ACHN** |  |  |  |  |
|  | POMHEX | 0.326 | 0.166 | 0.100 |
|  | 5MPN | 65.745 | 21.888 | 8.402 |
|  | PIK-III | 2.033 | 1.040 | 0.692 |
|  | Afatinib | 8.932 | 2.752 | 0.446 |
| **A498** |  |  |  |  |
|  | POMHEX | 0.307 | 0.094 | 0.031 |
|  | 5MPN | 3.541 | 0.752 | 0.080 |
|  | PIK-III | 4.173 | 3.004 | 2.380 |
|  | Afatinib | 5.524 | 3.751 | - |

SI Methods

**Microscopy for immunofluorescence and probe**

A498 cells were seeded in 12-well plate, and the appropriate size of cell crawler was placed in advance, 20 thousand cells per well. MEM complete medium was added and placed in the incubator, and each well was treated accordingly after 48 hours. The medium was aspirated out of the wells after 8 hours, and 200 nM Mitotraker-red (M7512, ThermoFisher) was added to the wells. Mitochondrial staining was performed for 30 min, then paraformaldehyde fixation for 15 min, and indirect immunofluorescence was performed in fixed cells by incubation with DLAT antibody (12362S, CST, dilution 1:100) overnight at 4°C, followed by incubation with Alexa Fluor 488 anti-mouse secondary antibody for 1 hour at room temperature. Cells were incubated with Hoechst 33342 (4082S, CST, 5μg/ml dilution) for 15 minutes at room temperature. Cell crawls were photographed under Confocal (LSM880, Zeiss, Germany). Images were processed using Image J.

**LC3 and Nile red staining:** OSRC-2 cells were seeded in 12-well plate crawls, and each well was treated accordingly after 48 hours. After 8 hours, the medium in the wells was aspirated, washed with PBS, and then cells were fixed with paraformaldehyde for 15 min. The cells were incubated with LC3 antibody (14600-1-AP, Proteintech, Dilution:1:200) at 4°C overnight, then incubated with Alexa Fluor 488 anti-rabbit secondary antibody for 1 hour at room temperature for indirect immunofluorescence. After washing, Nile red-hoechst dye was added (Nile red 1:40, hoechst 5μg/ml, dissolved in ultrapure water), and incubated for 20 min at 37°C. After the PBS wash, Confocal (Zeiss. LSM880, Germany) was used to photograph the cell crawls.

**JC-1/mitoSOX probe staining (MT09, MT14, Dojindo):** 20 thousand A498 cells per dish were cultured for 48 hours and then treated with the appropriate drugs for 8 hours. JC-1 staining: HBSS washed with residual drugs, 4uM JC-1 working solution was required, and incubated for 30 min at 37°C in an incubator. Supernatants were removed, and the cells were washed twice with HBSS. MitoSOX staining: 1000-fold dilution of 10 mmol/l mtSOX Deep Red DMSO stock solution to make 10 μmol/l working solution (mitoSOX 1:1000, Hoechst 5ug/ml, HBSS dilution). Add the prepared mitoSOX Deep Red working solution, and incubate at 37°C for 30 min. After washing with HBSS for 3 times, the probe was stabilized with Image buffer solution and observed under confocal microscope.

**Oxygen Consumption Rate (OCR) and extracellular acidification rate (ECAR)**

OCR and ECAR measurements using seahorse cellular flux assays. A498 cells were plated with 1× 10^4^ cells per XF96 well to ensure 90% surface coverage at the time of the experiment after 12 h of drug treatment. For OCR analysis, medium was exchanged for mitochondrial stress medium (Seahorse XF Assay Medium supplemented with 2 mM glutamine and 10 mM glucose) at 1 hour before the assay. The cultures were similarly equilibrated in non-CO_2_ incubator. Substrates and selective inhibitors were injected to achieve final concentrations of oligomycin (Sigma-Aldrich 495455) at 1.0 μM, carbonyl cyanide 4-(trifluoromethoxy) phenylhydrazone (FCCP; Sigma-Aldrich, C2920) at 0.5 and 1 μM, and rotenone-antimycin A (Sigma-Aldrich 557368 and A8674) at 0.5 μM, according to the manufacturer’s instructions. For glycolysis analyses, medium was exchanged for Seahorse Glycolysis Stress medium (Seahorse XF Assay Medium supplemented with 2 mM glutamine) at 1 h before the assay. The cultures were similarly equilibrated in non-CO_2_ incubator. Substrates and selective inhibitors were injected to achieve final concentrations of glucose at 10 mM, oligomycin at 1.5 μM, and 2-deoxy-D-glucose (2-DG; Medchemexpress, HY-13966) at 50 mM, according to the manufacturer’s instructions. The OCR and ECAR values were further normalized to the number of cells present in each well.

**Fluorescence detection of macropinocytosis**

Elesclomol and/or PIK-III were treated with FITC-dextran for 8 hours, then medium containing FITC-dextran and mitotraker or lysotraker was added and observed by confocal microscopy. FITC-dextran was used at a concentration of 5 μM, lysotraker was used at a concentration of 100 nM, and mitotraker deep red was used at a concentration of 500 nM. Mitotraker deep red was used at a concentration of 500 nM. FITC-dextran (HY-128868, MCE Chem), Lysotraker (40739ES50, Yeasen), Mitotraker (M22426, ThermoFisher).

**Immunohistochemistry**

Immunohistochemistry (IHC) was performed following the instructions of the SABC anti-rabbit-POD kit (SA1028, Boster Bioengineering). Paraffin sections were dewaxed with gradient ethanol, steam heated for antigen retrieval in EDTA-based buffer, blocked with 3% H_2_O_2_ followed by 5% BSA. The specimens were then incubated with primary antibody at 4°C overnight, rewarmed to 37°C for 1 hour and washed with PBS. Subsequently, tissue slices were exposed to drop-wise additions of biotinylated IgG as secondary antibodies for 30 min at 37°C. After incubation with SABC substance for 30 min at 37°C, DAB was used for color reaction. Slides were counterstained with hematoxylin, mounted, and imaged under a High-Resolution Digital Pathology Slice Scanning System. Information of primary antibodies: FDX1(12592-1-AP, Proteintec), Ki-67(ab279653, abcam), phos-PDHA1(ab177461, abcam), phos-PDHK (11596, Signal Way), TPK1(10942-1-AP, Proteintech).

**Enzyme Activity Assay**

PDH Enzyme Activity Microplate Assay Kit (AB109902, abcam): **Prepare Sample**: Bring sample (A498/OSRC-2 cells treated as mentioned) to appropriate concentration in PBS [15 mg/ml]. Perform detergent extraction with appropriate amount of Detergent [1/10 for cultured cells]; **Load Plate：**Load samples on plate being sure to include positive control sample and buffer control as a null reference. Incubate 3 hours at room temperature; **Measure：**Rinse wells twice with stabilizer. Make sufficient Assay Solution to load 200 μl/well. Add 200 μL Assay Solution into each well. Measure OD 450 nm at 20 second intervals for up to 15 minutes. Hydroxyethyl TPP assay was performed following instruction (BC0385, Soluble).

**Vitamin B1 assay** (BC4195, Solarbio)**:**

Add 0.6mL of extraction solution per 5 million cells, and ultrasonically break the cells in ice bath; add 0.4mL of distilled water, mix well and centrifuge at 13000g at 25°C for 10min, and take the supernatant for determination. The 10mg/mL standard solution was diluted to 250, 125, 62.5, 31.25, 15.625, 7.8125 μg/mL standard solution. After 25μl Standard solution was added into 975μl dilution solution, 200μl diluted standard solution was mixed in another 200μl dilution solution. Proportion the test reagents evenly. Mix well, 80 degrees Celsius water bath for 30min, add 200μL in 96-well plate to determine the absorbance value at 704nm.

**PDX Construction**

The current study protocol was approved by the Animal Ethics Committee of the Fifth Affiliated Hospital of Sun Yat-sen University (FH-SYS 2025-109). The tumor samples were collected with informed consent from all participants. Six to-eight-week-old NSG mice originally obtained from the SPF (Beijing) Biotechnology were implanted subcutaneously with patient-derived TGs. After the establishment of tumorgrafts, calipers were used to measure tumor dimensions. Volume was calculated by multiplying width, length, and height, thereby minimizing the potential bias of disproportionately weighing one particular dimension (at the cost of overestimating tumor volumes). Once tumor volumes reached ~100-200mm^3^, mice were treated with ES/PIK-Ⅲ or vehicle as a control. Tumors were measured weekly. As indicated, tumors were harvested around 35 days.

**3D-TSCs**

Human kidney cancer tissues were obtained from Department of Urology, The Fifth Affiliated Hospital of Sun Yat-sen University (Clinical Tissue Sample Ethics Approval Number: K30-1-2022). vibrating slicer cut into equal portions of 300 μm per slice, tissue punch to take the diameter of 4 mm equal area of the tissue. Sliced tissues were placed in hydrogel culture, the outer layer of the medium to be F12 complete medium culture, for 2 days before the addition of drugs to treat the tumor tissue slices (elesclomol 100 nM, PIK-III 3 μM)

**Culture materials:** Solution A: CollagenⅠ; Solution B: 10×concentrated sterile culture medium (Ham^,^s F-12); Solution C: Sterile reconstitution buffer; Medium: Ham^,^s F-12 supplemented with 20% fetal bovine serum and 50 μg/ml gentamicin reagent solution(10 mg/ml in distilled water).

**Methods:** Add ice-cold solution B to A at a volume ratio 1:8 and add mix well in a 50 ml conical tube on ice; Add 1 volume of ice-cold solution C to the mixture of A and B. Mix well on ice until the color of the mixture turns pink; Pour 1 ml of reconstituted collagen solution into each 30 mm diameter Millicell insert in a tissue culture hood. Leave inserts in the hood at room temperature for 20-30 min until collagen solidifies completely. To expedite solidification, inserts with collagen can be placed in a 37°C incubator. Pipette the minced tissue on collagen gel. Pour 0.5 ml of collagen gel onto the inner dish with bottom layer gel. A 0.5 cm length of murine gastrointestinal tissue is enough to prepare 1 ml of tissue-collagen gel mixture. Transfer the covered outer dish to a 37°C incubator and allow the gel of the inner dish to solidify for 20-30 min. After solidifying of the top layer tissue-containing gel, add 1.5 ml culture media into the outer dish in the hood.

**MTT assay:** Tumor slices with same size using tissue perforator were seeded on collagen gel-coated inserts in 24-well plates. After drug treatment for 96 hours, 3D-TSCs were collected and enzymatically dissociated at 37°C using Digestion I (10 mg/ml insulin, 10 mg/ml hydrocortisone, 100 μg/ml EGF, 1.2 mg/ml collagenase III, 0.02 mg/ml hyaluronidase in DMEM/Hams F12) for 2 hours and followed by digestion II (5 mg/ml dispase II, 0.1 mg/ml DNAase I, 20 mM HEPES in DMEM/Hams F12) for 5 min. Cells were filtered through a 0.40 μM strainer and pelleted, resuspended in PBS buffer. Cells were mixed with an equal volume of trypan blue.

**Staining of live dead cells (Dojindo, C542):** Preparation of 1 mmol/l Calcein-AM storage solution: add 1ml DMSO to a tube containing 1 mg Calcein-AM powder. Preparation of staining working solution: restore Calcein-AM storage solution and PI storage solution to room temperature and use. Add 10 ul of Calcein-AM storage solution and 15ul of PI storage solution to 5 ml of PBS and mix well to make working solution. The concentration of Calcein-AM was 2 mol/l, while the concentration of PI was 4.5 mol/l. Staining steps: Remove the culture medium; Wash the inner chambers with PBS for 3 times, and absorb the PBS; Add staining working solution to each chamber, and incubate at 37°C for 30 min; Observe the yellow-green fluorescent live cells and the red fluorescent dead cells at the same time under the excitation wavelength of 490± 10 nm. Observe the dead cells alone with 545 nm excitation wavelength by fluorescence somatoscopic microscopy (Nikon, SMZ18).

**Electron microscopy**

Main instruments: ultrathin sectioning machine (Leica UC7, Germany), diamond sectioning knife (Diatome Ultra 45°), transmission electron microscope (Hitachi HT7800, Japan). Experimental steps: collect 10 million cells for each group, immediately fix them into 1.5 ml centrifuge tubes with 2.5% glutaraldehyde, and fix them in the refrigerator for 12-24 hours. Embedding and polymerization: fix the samples in 1% osmiic acid + 2% potassium ferricyanide mixture for 1-2 hours. Carefully remove the osmium acid waste solution and rinse with 18.25 MΩ deionized water for 4 times, 15 minutes each time. For gradient dehydration, samples were sequentially added to 30%, 50%, 70%, 80% and 95% acetone for 10 min each time, and finally 100% acetone for 20 min. For osmotic embedding, acetone: embedder=3:1 37°C for 1 hour, acetone: embedder=1:1, 37°C for 3 hours, and pure embedding agent 37°C overnight. The samples were baked in a 60°C oven for 48 hours, and the resin block was removed and set aside. Ultrathin slicer cut the resin block into 70-90 nm ultrathin slices, and then fished with copper mesh. Staining with uranyl acetate for 8-15 minutes and lead citrate for 8-10 minutes, drying and waiting for detection. Transmission electron microscope was photographed and observed.

**Transcriptomics**

A498 was chosen for transcriptome and untargeted metabolome sequencing because it demonstrated consistent responses to the treatment combinations in vitro. Under hypoxic conditions, A498 required a much lower concentration of PIK-III for cuproptosis sensitization(1µM) than that required by ACHN(3µM). In addition, to ensure consistency in the conditions and purpose of compound library screening, the A498 cell line with a well-defined VHL mutation and activation of the HIF pathway was more compatible with the model cells producing the warburg effect.

1. RNA extraction Total RNA was extracted from the treated OSRC2 cells using TRIzol® Reagent according the manufacturer’s instructions (Magen). RNA samples were detected based on the A260/A280 absorbance ratio with a Nanodrop ND-2000 system (Thermo Scientific, USA), and the RIN of RNA was determined by an Agilent Bioanalyzer 4150 system (Agilent Technologies, CA, USA). Only

qualified samples will be used for library construction.

2. Library preparation and Sequencing Paired-end libraries were prepared using a ABclonal mRNA-seq Lib Prep Kit (ABclonal, China) following the manufacturer’s instructions. The mRNA was purified from 1 μg total RNA using oligo(dT) magnetic beads followed by fragmentation carried out using divalent cations at elevated temperatures in ABclonal First Strand Synthesis Reaction Buffer. Subsequently, first-strand cDNAs were synthesized with random hexamer primers and Reverse Transcriptase (RNase H) using mRNA fragments as templates, followed by second-strand cDNA synthesis using DNA polymerase I, RNAseH, buffer, and dNTPs. The synthesized double stranded cDNA fragments were then adapter-ligated for preparation of the paired-end library. Adaptor-ligated cDNA was used for PCR amplification. PCR products were purified (AMPure XP system) and library quality was assessed on an Agilent Bioanalyzer 4150 system. Finally, the library preparations were sequenced on an Illumina Novaseq 6000 (or MGISEQ-T7) and 150 bp paired-end reads were generated.

**3.Data analysis**

The data generated from Illumina (or BGI) platform were used for bioinformatics analysis. All of the analyses were performed using an in-house pipeline from Shanghai Applied Protein Technology. The major software and parameters are as follows.

**3.1 Quality control**

Raw data (or Raw reads) of fastq format were firstly processed through in-house perl scripts. Inthis step, remove the adapter sequence and filter out low quality (low quality, the number of lines with a string quality value less than or equal to 25 accounts for more than 60% of the entire reading) and N (N means that the base information cannot be determined) ratio is greater than 5% reads to obtain clean reads that can be used for subsequent analysis.

**3.2 Mapping**

Then clean reads were separately aligned to reference genome with orientation mode using HISAT2 software (http://daehwankimlab.github.io/hisat2/) to obtain mapped reads.

**3.3 Quantification of gene expression level**

FeatureCounts (http://subread.sourceforge.net/) was used to count the reads numbers mapped to each gene. And then FPKM of each gene was calculated based on the length of the gene and reads count mapped to this gene.

**3.4 Differential expression analysis**

Differential expression analysis was performed using the DESeq2

(<http://bioconductor>.org/packages/release/bioc/html/DESeq2.html), DEGs with |log2FC|> 1 and *P*-adj< 0.05 were considered to be significantly different expressed genes.

**3.5 Enrichment analysis**

The GO and KEGG enrichment analysis of differential genes can explain the functional enrichment of differential genes and clarify the differences between samples at the gene function level. We use clusterProfiler R software package for GO function enrichment and KEGG pathway enrichment analysis. When *P*< 0.05, it is considered that the GO or KEGG function is significantly enriched.

**3.6 PPI analysis**

PPI analysis is used to study whether there is interaction between gene products--proteins. The analysis is based on the protein information corresponding to genes. Based on the STRING database (https://www.string-db.org/), which known and predicted Protein-Protein Interactions. For the species existing in the database, we construct the networks by extract the target gene list from the database. For species not included in the database, the target gene set sequence is first aligned with the reference sample protein sequence contained in the STRING protein interaction database with blastx, and the protein interaction relationship of the reference species is used to establish an interaction network.

**Trace elements assay**

Trace element extraction: Take 50 μL of the sample to be tested, put it into a 15 mL centrifuge tube, add 2.45 mL of sample dilution liquid and mix it well. After sufficient inversion and mixing, the sample was injected into the ICP-MS autosampler for analysis, and the needle wash solution was analyzed by 1% nitric acid water mass spectrometry; Data Acquisition and Analysis: The samples were analyzed by Inductively Coupled Plasma Mass Spectrometry (ICP-MS) using the NexION 1000G. The power (1600 w), nebulizer gas flow rate of 1.08 L/M, auxiliary gas flow rate of 1.2 L/M, plasma gas flow rate of 15 L/M, the number of scans was 3, and the total acquisition time was 4.5 min. The data acquisition and processing were carried out by Syngistix (version2.4), and quantitative analysis was performed by the internal standard method.

**Metabolomics**

**Metabolite Extractions:** The culture medium from the cultured cells (10^7 cells per sample) was removed using pipette. Then the cells were washed with PBS under 37°C and the PBS was removed. 800 μL of cold methanol/acetonitrile (1:1, v/v) was added to remove the protein and extract the metabolites. The mixture was collected into a new centrifuge tube, and centrifuged at 14000 g for 5 min at 4°C to collect the supernatant. The supernatant was dried in a vacuum centrifuge. For LC-MS analysis, the samples were re-dissolved in 100 μL acetonitrile/water (1:1, v/v) solvent.

**LC-MS Analysis:** For untargeted metabolomics of polar metabolites, extracts were analyzed using a quadrupole time-of-flight mass spectrometer (Sciex TripleTOF 6600) coupled to hydrophilic interaction chromatography via electrospray ionization in Shanghai Applied Protein Technology Co., Ltd. LC separation was on a ACQUIY UPLC BEH Amide column (2.1 mm × 100 mm, 1.7µm particle size (waters, Ireland) using a gradient of solvent A (25 mM ammonium acetate and 25 mM ammonium hydroxide in water) solvent B (acetonitrile). The gradient was 85% B for 1 min and was linearly reduced to 65% in 11 min, and then was reduced to 40% in 0.1 min and kept for 4 min, and then increased to 85% in 0.1 min, with a 5 min re-equilibration period employed. Flow rate was 0.4 mL/minute, column temperature was 25 °C, auto sampler temperature was 5°C, and injection volume was 2 µL. The mass spectrometer was operated in both negative ion and positive ionizations mode. The ESI source conditions were set as follows: Ion Source Gas1 (Gas1) as 60, Ion Source Gas2 (Gas2) as 60, curtain gas (CUR) as 30, source temperature: 600℃, IonSpray Voltage Floating (ISVF) ± 5500 V. In MS acquisition, the instrument was set to acquire over the m/z range 60-1000 Da, and the accumulation time for TOF MS scan was set at 0.20 s/spectra. In auto MS/MS acquisition, the instrument was set to acquire over the m/z range 25-1000 Da, and the accumulation time for product ion scan was set at 0.05 s/spectra. The product ion scan is acquired using information dependent acquisition (IDA) with high sensitivity mode selected. The parameters were set as follows: the collision energy (CE) was fixed at 35 V with ± 15 eV; declustering potential (DP), 60 V (+) and -60 V (-); exclude isotopes within 4 Da, candidate ions to monitor per cycle: 10.

**Data Analysis:** The raw MS data (wiff.scan files) were converted to MzXML files using ProteoWizard MSConvert before importing into freely available XCMS software. For peak picking, the following parameters were used: centWave m/z = 25 ppm, peakwidth = c (10, 60), prefilter = c (10, 100). For peak grouping, bw = 5, mzwid = 0.025, minfrac = 0.5 were used. In the extracted ion features, only the variables having more than 50% of the nonzero measurement values in at least one group were kept. Compound identification of metabolites by MS/MS spectra with an in-house database established with available authentic standards. After normalized to total peak intensity, the processed data were uploaded into before importing into SIMCA-P (version 14.1, Umetrics, Umea, Sweden), where it was subjected to multivariate data analysis, including Paretoscaled principal component analysis (PCA) and orthogonal partial least-squares discriminant analysis (OPLS-DA). The 7-fold cross-validation and response permutation testing was used to evaluate the robustness of the model. The variable importance in the projection (VIP) value of each variable in the OPLS-DA model was calculated to indicate its contribution to the classification. Significance was determined using an unpaired Student’s t test. VIP value > 1 and *P*< 0.05 was considered as statistically significant.

**Bioinformatics Analysis:** For KEGG pathway annotation, the metabolites were blasted against the online Kyoto Encyclopedia of Genes and Genomes (KEGG) database to retrieve their Cos and were subsequently mapped to pathways in KEGG11. The corresponding KEGG pathways were extracted. To further explore the impact of differentially expressed metabolites, enrichment analysis was performed. KEGG pathway enrichment analyses were applied based on the Fisher’ exact test, considering the whole metabolites of each pathway as background dataset. And only pathways with *p*-values under a threshold of 0.05 were considered as significant changed pathways. For hierarchical clustering, Cluster 3.0 (<http://bonsai.hgc.jp/~mdehoon/software/cluster/software.htm>) and the Java Treeview software (http://jtreeview.sourceforge.net) were used. Euclidean distance algorithm for similarity measure and average linkage clustering algorithm (clustering uses the centroids of the observations) for clustering were selected when performing hierarchical clustering. Heat map is often presented as a visual aid in addition to the dendrogram.
